# The DNA methylation landscape of the root‐knot nematode‐induced pseudo‐organ, the gall, in Arabidopsis, is dynamic, contrasting over time, and critically important for successful parasitism

**DOI:** 10.1111/nph.18395

**Published:** 2022-09-02

**Authors:** Ana Cláudia Silva, Virginia Ruiz‐Ferrer, Sebastian Y. Müller, Clement Pellegrin, Patricia Abril‐Urías, Ángela Martínez‐Gómez, Almudena Gómez‐Rojas, Eduardo Berenguer, Pilar S. Testillano, Maria Fe Andrés, Carmen Fenoll, Sebastian Eves‐van den Akker, Carolina Escobar

**Affiliations:** ^1^ Facultad de Ciencias Ambientales y Bioquímica Universidad de Castilla‐La Mancha Área de Fisiología Vegetal, Avda. Carlos III, s/n 45071 Toledo Spain; ^2^ Department of Plant Sciences University of Cambridge Cambridge CB2 3EA UK; ^3^ Centro de Investigaciones Biológicas Margarita Salas CIB‐CSIC, Pollen Biotechnology of Crop Plants Ramiro de Maeztu 9 28040 Madrid Spain; ^4^ Instituto de Ciencias Agrarias (ICA, CSIC) Protección Vegetal, Calle de Serrano 115 28006 Madrid Spain; ^5^ International Research Organization for Advanced Science and Technology (IROAST) Kumamoto University Kumamoto 860‐8555 Japan

**Keywords:** Arabidopsis, DNA methylation/epigenetics signatures, galls, giant cells, *Meloidogyne javanica*, siRNAs, tomato, transposons

## Abstract

- Root‐knot nematodes (RKNs) induce giant cells (GCs) within galls which are characterized by large‐scale gene repression at early stages. However, the epigenetic mechanism(s) underlying gene silencing is (are) still poorly characterized.
- DNA methylation in Arabidopsis galls induced by *Meloidogyne javanica* was studied at crucial infection stages (3 d post‐infection (dpi) and 14 dpi) using enzymatic, cytological, and sequencing approaches. DNA methyltransferase mutants (*met1*, *cmt2*, *cmt3*, *cmt2/3*, *drm1/2*, *ddc*) and a DNA demethylase mutant (*ros1*), were analyzed for RKN resistance/tolerance, and galls were characterized by confocal microscopy and RNA‐seq.
- Early galls were hypermethylated, and the GCs were found to be the major contributors to this hypermethylation, consistent with the very high degree of gene repression they exhibit. By contrast, medium/late galls showed no global increase in DNA methylation compared to uninfected roots, but exhibited large‐scale redistribution of differentially methylated regions (DMRs). In line with these findings, it was also shown that DNA methylation and demethylation mutants showed impaired nematode reproduction and gall/GC‐development. Moreover, siRNAs that were exclusively present in early galls accumulated at hypermethylated DMRs, overlapping mostly with retrotransposons in the CHG/CG contexts that might be involved in their repression, contributing to their stability/genome integrity. Promoter/gene methylation correlated with differentially expressed genes encoding proteins with basic cell functions. Both mechanisms are consistent with reprogramming host tissues for gall/GC formation.
- In conclusion, RNA‐directed DNA methylation (RdDM; DRM2/1) pathways, maintenance methyltransferases (MET1/CMT3) and demethylation (ROS1) appear to be prominent mechanisms driving a dynamic regulation of the epigenetic landscape during RKN infection.

## Introduction

Plant‐parasitic nematodes cause severe losses in agriculture (Singh *et al*., 2015). The endo‐parasitic root‐knot nematodes (RKN; *Meloidogyne* spp.) are among the major contributors. Root‐knot nematodes penetrate host roots, delivering effectors into the vascular cylinder and inducing the development of a new organ (several giant cells; GCs) housed within a gall, from which they feed (Escobar *et al*., 2011, 2015). The multinucleated GCs undergo mitosis with incomplete cytokinesis (Escobar *et al*., 2015).

Several studies have reported observations of generalized gene repression in Arabidopsis and tomato galls, particularly in isolated GCs at early infection stages (Jammes *et al*., 2005; Fuller *et al*., 2007; Barcala *et al*., 2010; Portillo *et al*., 2013). However, the underlying mechanisms are poorly understood.

Recent studies point towards a role for epigenetic regulation of gene expression during the organogenesis of galls/GCs – several microRNAs (miRNAs; e.g. miR390, miR159, miR172) involved in plant developmental processes in Arabidopsis are crucial for gall/GC formation (Cabrera *et al*., 2016; Medina *et al*., 2017; Díaz‐Manzano *et al*., 2018; reviewed in Cabrera *et al*., 2018b; Jaubert‐Possamai *et al*., 2019; Hewezi, 2020). In addition, large‐scale sequencing of small RNAs (sRNAs) has revealed the accumulation of 24‐nucleotide (nt) small‐interfering RNAs (siRNAs) at early (3 d post‐infection; dpi; Cabrera *et al*., 2016) and medium/late (7 dpi; Medina *et al*., 2017) infection stages in Arabidopsis. The abundance of such sRNAs, corresponding to repetitive regions of the genome (repeat associated small‐interfering RNAs; rasiRNAs; Cabrera *et al*., 2016; Ruiz‐Ferrer *et al*., 2018), is a gall hallmark. Interestingly, gall‐distinctive rasiRNAs accumulated differentially in transposable elements (TEs), and, accordingly, major members of retrotransposon superfamilies (COPIA, GYPSY, LINE and SINE) were repressed in early galls (Ruiz‐Ferrer *et al*., 2018). Consistent with this, Medina *et al*. (2018) identified siRNA clusters that were differentially expressed in Arabidopsis galls (7, 14 dpi), and overrepresentation of 23–24‐nt siRNAs.

sRNAs induce either post‐transcriptional gene silencing (PTGS) by targeting complementary mRNAs for degradation/translational repression in the cytoplasm, or transcriptional gene silencing (TGS) by repressive epigenetic modifications, such as DNA methylation and histone modifications, to homologous regions of the genome (Ruiz‐Ferrer & Voinnet, 2009; Deleris *et al*., 2016). In plants, the major siRNA‐mediated epigenetic pathway is RNA‐directed DNA methylation (RdDM; Matzke *et al*., 2015; Cuerda‐Gil & Slotkin, 2016). Plant DNA methylation occurs in all cytosine contexts: CG, CHG and CHH (where H is A, C or T). The establishment of *de novo* DNA methylation is catalyzed by DOMAINS REARRANGED METHYLASE 2 (DRM2) via the RdDM pathway (Ye *et al*., 2016), while maintenance of DNA methylation is mainly mediated by METHYLTRANSFERASE1 (MET1), CHROMOMETHYLASE 3 (CMT3) and DRM2 or CMT2 in the CG, CHG and CHH methylation contexts, respectively (Zhang *et al*., 2018). Plant genomes also encode DNA glycosylases/lyases that remove cytosine methylation (Ortega‐Galisteo *et al*., 2008), including REPRESSOR OF SILENCING 1 (ROS1; Gong *et al*., 2002; Zhu *et al*., 2007). Changes in DNA methylation were reported in soybean–cyst nematode and Arabidopsis–cyst nematode interactions, and in galls formed by *Meloidogyne graminicola* in rice (Rambani *et al*., 2015; Hewezi *et al*., 2017; reviewed in Hewezi, 2020; Atighi *et al*., 2020; respectively). However, a role for DNA methylation and its interplay with the dynamic regulation of sRNAs in the control of gene expression in galls of dicotyledonous species has not been described.

In this study, we analyzed the differential methylome of galls induced by *Meloidogyne javanica* at two critical infection time points (3 dpi and 14 dpi) from early gall formation to medium/late infection stages, showing a dynamic regulation of the epigenetic landscape. Immunolocalization of methylation marks also showed the predominant contribution of the GCs at 3 dpi, among different cell types within the gall. The functions of several methylases involved in the RdDM pathway (such as DRM2), as well as those involved in methylation maintenance (CMT3, CMT2, MET1), together with DNA demethylase ROS1, are discussed in the context of gall formation. Our results strongly suggest that DNA methylation mediated by DRM2/1 through RdDM pathways and by maintenance methylases may contribute to preservation of genome integrity, while dramatic reprograming processes drive GC and gall formation.

## Materials and Methods

### Plant lines, nematode maintenance, reproduction tests and giant cell phenotyping

*Meloidogyne javanica* (Treub, 1885) Chitwood, 1949 populations were maintained *in vitro* on cucumber (*Cucumis sativus* L. cv. Johanna) roots (Díaz‐Manzano *et al*., 2016). *Arabidopsis thaliana* (L.) Heynh. Columbia‐0 (Col‐0) seeds were sterilized, grown, and infected as described by Olmo *et al*. (2017). Galls and uninfected root segments (RCs) were hand‐dissected at 3 and 14 dpi (counting from 24 h after inoculation) following Barcala *et al*. (2010).

*Solanum lycopersicum* L. cv. Moneymaker (Buzzy^®^ seeds, Heerenveen, Holland, cat. no. 002845) seeds were grown *in vitro* and inoculated as in Portillo *et al*. (2013). Tomato galls and uninfected root segments (i.e. controls; RCs) were hand‐dissected at early stages of infection (from 2 to 7 dpi; Portillo *et al*., 2013).

Homozygous Arabidopsis T‐DNA insertion lines and the primers used for PCR genotyping are given in Supporting Information Table S1. For *in vitro* nematode infection tests, at least three independent biological replicates were used for each mutant: *met1‐3*, *cmt2‐3*, *cmt2‐3/cmt3‐11*, *cmt3‐11*, *drm1‐2drm2‐2*, *ddc* and *ros1* (≥ 111 plants per line) vs Col‐0. Seeds were sterilized and grown as described for Col‐0. Gall diameter was measured at 14 dpi (*n* ≥ 25 per line tested) using the measurement tools in Fiji (Schindelin *et al*., 2012).

Reproductive parameters were determined in soil‐grown plants: number of females, number of egg masses produced, and number of total eggs per line (Olmo *et al*., 2020), in at least seven independent biological replicates per line (Student's *t*‐test; *P* < 0.05). Giant cell phenotyping was carried out as described by Cabrera *et al*. (2018a).

### RNA and DNA extraction and purification

Collection of Arabidopsis plant material and total RNA and genomic DNA extraction from the same samples were performed as described by Silva *et al*. (2019). Further details are provided in Methods S1.

### Quantification of global 5‐methylcytosine (5‐mC)

To compare levels of global DNA methylation in galls induced by *M. javanica* in Arabidopsis and tomato with those in their corresponding RCs, the MethylFlash™ Global DNA Methylation (5‐mC) ELISA Easy Kit was used (EpiGentek, New York, NY, USA) with *c*. 100 ng of DNA for each of the three independent biological samples for Arabidopsis (see the previous section). In tomato, a total of seven independent experiments were pooled in four independent biological samples per treatment. Each independent biological replicate contained at least 50 galls and > 30 RCs (DNA was extracted according to the procedure described by Silva *et al*., 2019), together with the negative and positive DNA controls provided in the kit. The 5‐mC percentage was calculated according to the manufacturer's instructions.

### Immunofluorescence and confocal microscopy

To localize 5‐mC in GCs within gall sections, we performed immunofluorescence assays on resin sections (Testillano *et al*., 2013), followed by confocal analyses. 5‐methylcytosine immunofluorescence was quantified using Fiji v.2.0.0‐rc69/1.52n (Schindelin *et al*., 2012). The maximum projections (*n* ≥ 20 optical sections) of the 4′,6‐diamidino‐2‐phenylindole (DAPI) channel were used to define the Regions of Interest (ROIs) corresponding to the individual nuclei, and the maximum projections of the 5‐mC (green) channel were used to measure fluorescence intensity values within ROIs. Only the ROIs presenting a maximum value of 85 (green channel) were considered. The corrected fluorescence intensity of each ROI was calculated as follows: Integrated density – (Area of selected nuclei × Mean fluorescence of background readings (adapted from Gavet & Pines, 2010; McCloy *et al*., 2014)). The average of the background readings was obtained using the mean fluorescence of two squares drawn in the background of each image. Significant differences were calculated using the Kruskal–Wallis test followed by Dunn's post hoc test (*P* < 0.05).

### High‐throughput MethylC‐sequencing (MethylC‐seq) library preparation and data processing

The six independent MethylC‐seq libraries, three for galls and three for RC at 3 dpi (see Table S2 for further details), were prepared using the NEXTflex^®^ Bisulfite‐Seq Library Prep Kit (Bioo Scientific, Austin, TX, USA), indexed for post‐sequencing demultiplexing, pooled in equimolar amounts and sequenced in one lane of an Illumina^®^ HiSeq 4000 PE100 platform (San Diego, CA, USA) and Illumina^®^ HiSeq X PE150 (RC2 and RC3; San Diego, CA, USA) by AllGenetics & Biology SL, A Coruña, Spain.

The six independent MethylC‐seq libraries, three for galls and three for RC at 14 dpi (see Table S2 for further details), were prepared using the EZ DNA Methylation‐Gold Kit (Zymo Research, Freiburg, Germany) following the library protocol Accel‐NGS Methyl‐Seq DNA Library Kit for Illumina Platforms by Macrogen Inc. (Seoul, South Korea) and sequenced using an Illumina^®^ HiSeq X PE150.

MethylC‐seq data were processed using the pipeline available at https://github.com/seb‐mueller/snakemake‐bisulfite (commit f12bb6) in GitHub (GEO accession numbers GSE155853, GSE156025). To identify differentially methylated regions (DMRs), we used MethylKit v.0.99.2 (Akalin *et al*., 2012) with the adjusted *P*‐value (*q*‐value) < 0.05. We defined regions in the form of adjacent non‐overlapping windows of 200 base pairs (bp; referred to hereafter as ‘bins’). Bins were annotated as those overlapping with genes, promoters, or transposable elements (TEs). ‘Promoter/TE’ hereafter refers to a DMR that overlaps a gene promoter and a TE (as specified in Araport11, 06/2016; Cheng *et al*., 2017). Promoters are considered regions from 1000 bp upstream to 200 bp downstream of the transcription start point. For each region, the methylated and unmethylated cytosines were counted (separately for each library/region/methylation context) and tested for differential methylation through a logistic regression approach using the ‘MN’ overdispersion correction as implemented in MethylKit. Several DMRs were validated (Methods S1; Fig. S1d).

### High‐throughput RNA‐sequencing (RNA‐seq) library preparation and data processing

Illumina's TruSeq Stranded mRNA Library Prep Kit was used to prepare the six independent libraries (three for galls and three for uninfected controls). Samples were dual‐indexed for post‐sequencing demultiplexing, pooled in equimolar amounts and sequenced in a fraction of an Illumina^®^ HiSeq X PE150 by AllGenetics & Biology SL, A Coruña, Spain. A description of the DEGs acquired in TAIR10 and additional details are provided in Table S3.

RNA‐sequencing raw data and detailed data processing are available via GEO accession number GSE155171 and in Methods S1. Differential expression analysis was performed with deseq2 (v.1.20.0; Love *et al*., 2014) using R (v.3.5.2; R Core Team, 2018) and Rstudio (v.1.1.463; RStudio Team, 2018). Genes with a ¦log_2_‐fold change¦ > 0.5 and an adjusted *P*‐value < 0.01 were considered differentially expressed (DEGs).

The 3 dpi sRNA libraries (GEO accession number GSE71563) were generated as described by Cabrera *et al*. (2016) (Table S4; Methods S1).

## Results

### *Meloidogyne javanica* induces DNA hypermethylation during early establishment in Arabidopsis, but not at medium/late stages

**Fig. 1:**
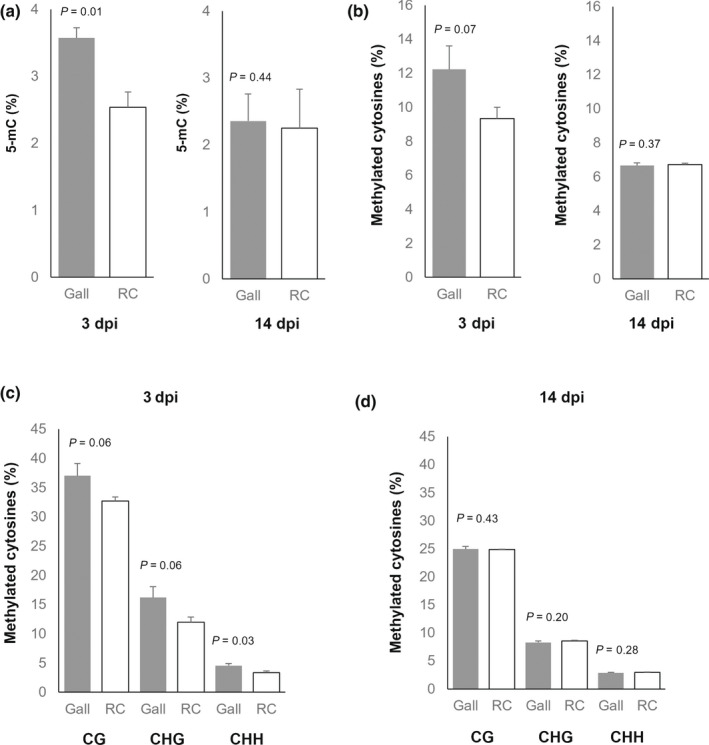
Global DNA methylation in Arabidopsis galls induced by *Meloidogyne javanica*. (a) Global 5‐methylcytosine (5‐mC) DNA methylation (in percentage terms) in galls and uninfected root segments (controls; RCs) at 3 and 14 d post‐infection (dpi), quantified using an ELISA‐based kit. (b) Global percentage of methylated cytosines (quantified using MethylC sequencing (MethylC‐seq)) in the same samples. (c, d) Global percentage of methylated cytosines (via MethylC‐seq) in galls at 3 dpi (c) and 14 dpi (d) and RCs in the three different methylation contexts (CG, CHG and CHH). Values are means from three independent biological samples (see Methods S1; ‘RNA and DNA extraction and purification’ sub‐section) and error bars denote ±SE. *P*‐values are indicated (one‐tailed Student's *t*‐test; *n* = 3).

Based on the very high degree of gene repression observed in GCs (Barcala *et al*., 2010), coupled with the differential abundance of siRNAs at early stages of gall formation (Cabrera *et al*., 2016; Ruiz‐Ferrer *et al*., 2018), we hypothesized that RdDM may play an important role in RKN feeding site formation. Therefore, we quantified the global 5‐mC DNA methylation induced by *M. javanica* in Arabidopsis at two infection stages, 3 and 14 dpi, which showed contrasting patterns of methylation, using two independent techniques: an ELISA‐based assay, and bisulfite sequencing/MethylC‐seq. The ELISA‐based approach identified significant differences in global DNA methylation at 3 dpi (Fig. 1a) compared to RCs, but not at 14 dpi (Fig. 1a). MethylC‐sequencing analysis of the same DNA showed a similar trend (*P* = 0.07; Fig. 1b; Table S2). The average degree of methylation in the CG, CHG, and CHH contexts was higher in 3 dpi galls than in RCs (Fig. 1c; Table S2c) with gall : RC ratios of 1.13, 1.35, and 1.38 respectively. The only significant difference between galls and RCs was found in the CHH context, although this context had the lowest level of DNA methylation (*P* = 0.03; Fig. 1c). By contrast, at 14 dpi, gall and RC global methylation levels were similar in all three methylation contexts (Fig. 1d; Table S2d). These data support the idea that there is hypermethylation in early galls, but there are no differences at 14 dpi galls, both compared with its correspondent RCs.

### DNA methylation preferentially targets transposable elements, particularly retrotransposons, in early galls

**Fig. 2:**
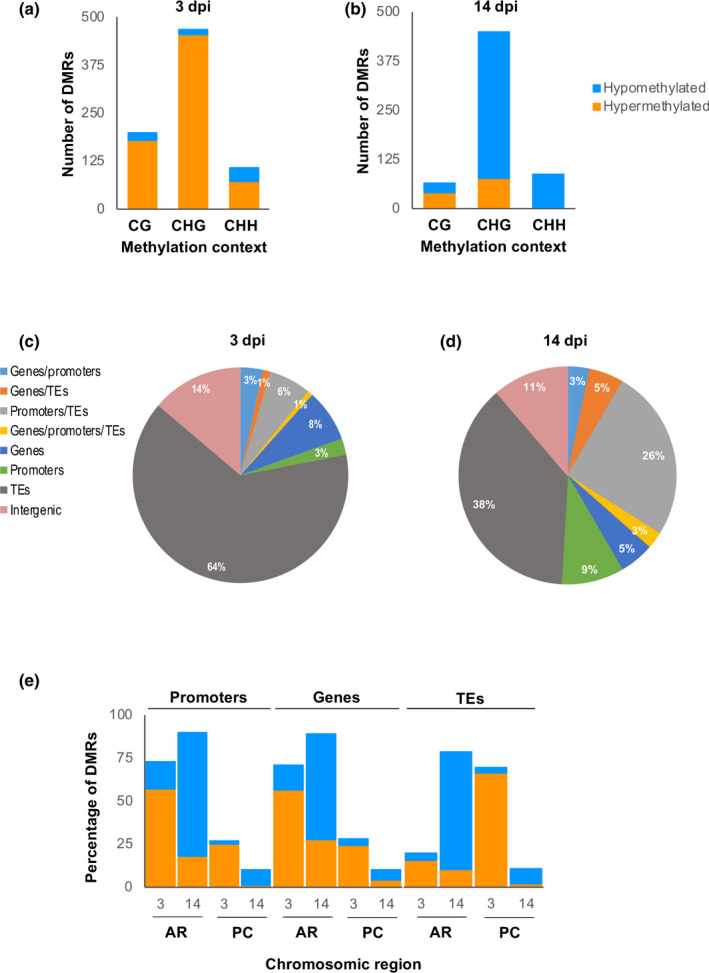
Distribution of differentially methylated regions (DMRs; methylation difference > 15%) in galls induced by *Meloidogyne javanica*. (a, b) Differentially methylated regions identified in the three methylation contexts (CG, CHG and CHH) at 3 d post‐infection (dpi) (a) and 14 dpi (b). (c, d) Percentages of different genomic regions (genes, promoters, transposable elements (TEs) and intergenic regions) overlapping with the identified DMRs at 3 dpi (c) and 14 dpi (d). (e) Classification of DMRs overlapping promoters, genes and TEs according to their chromosomic location at pericentromeric regions (PC) or chromosome arms (AR). In all cases, hypermethylation is shown in orange and hypomethylation in blue.

To identify DMRs of the genome that might be important for the interaction between Arabidopsis and RKNs at early and medium‐late infection stages, the Arabidopsis genome was divided into non‐overlapping regions of 200 bp. A total of 775 DMRs (minimum methylation difference 15%; Table S5) were identified at 3 dpi. Most DMRs were in the CHG context (199, 468, and 108 DMRs in the CG, CHG and CHH contexts, respectively; Fig. 2a). Consistent with our previous observations, the vast majority (*c*. 90%) of DMRs, in all contexts, were hypermethylated in early galls (Figs 1, 2). Further validation shows some representative DMRs matching five transposons belonging to different superfamilies (i.e. GYPSY, COPIA and LINE), a DNA transposon and a b‐ZIP transcription factor, all showing similar hypermethylation tendencies in 3 dpi galls with respect to their corresponding RCs (*q*‐value < 0.05) in both of the independent MethylC‐seq analyses (Fig. S1). Similarly, DMRs identified at 14 dpi (minimum methylation difference 15%; 603) were also predominant in the CHG context (65, 450 and 88 for CG, CHG and CHH, respectively). In contrast to the findings at 3 dpi, *c*. 80% of DMRs, in all contexts, were hypomethylated in 14 dpi galls (Fig. 2b; Table S5). Since there is no overall global change in the degree of methylation at 14 dpi (Fig. 1), this finding suggests a remodeling of the methylation landscape during the infection.

**Fig. 3:**
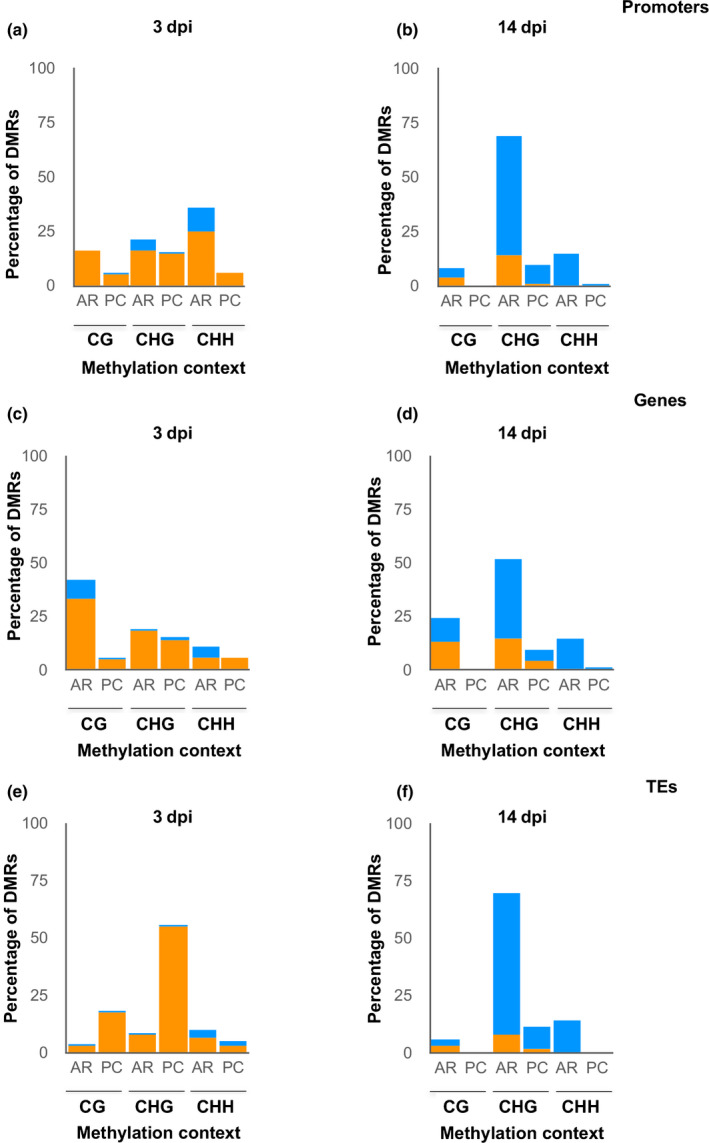
Classification of differentially methylated regions (DMRs; methylation difference > 15%) according to methylation contexts, chromosome location and genomic regions. (a–f) Classification of DMRs overlapping promoters (a, b), genes (c, d) and transposable elements (TEs) (e, f) according to their chromosomic location at pericentromeric regions (PC) or chromosome arms (AR), as well as by methylation context (CG, CHG or CHH) at 3 d post‐infection (dpi) (a, c, e) and 14 dpi (b, d, f). In all cases, hypermethylation is shown in orange and hypomethylation in blue.

Strikingly, at 3 dpi, the genomic elements which overlapped with most DMRs in all three cytosine contexts were transposable elements (TEs; 64%); far fewer DMRs overlapped with genes (8%) promoters (3%) and promoter/TEs (6%; Fig. 2c; Table S5). By contrast, a lower proportion of DMRs overlapped with TEs at 14 dpi, and the proportion of DMRs overlapping promoter/TEs was similar (38% and 26%; respectively; Fig. 2d; Table S5). Each Arabidopsis chromosome was categorized into two different regions: pericentromeric (PCs) and the rest of the chromosome arms (ARs; Ruiz‐Ferrer *et al*., 2018). At 3 dpi the vast majority of DMRs in promoters, genes and TEs were hypermethylated, independent of their position in the ARs or PCs (Fig. 2e). By contrast, by 14 dpi a large‐scale rearrangement of methylation is evidenced by the opposite pattern: the vast majority of DMRs in promoters, genes and TEs were hypomethylated, again, independent of their position in the ARs or PC (Fig. 2e). The results also show that at 3 dpi, while promoter and gene DMRs are located at ARs on the Arabidopsis genome with predominant CHH and CG contexts, respectively (Fig. 3a,c), DMRs overlapping TEs were mostly located at PC regions with a predominant CHG context (Fig. 3e). Interestingly, the pattern was also unequivocally different at 14 dpi, as DMRs overlapping TEs were mostly located in ARs and the predominant context in TEs, promoters and genes was CHG (Fig. 3b,d,f).

**Fig. 4:**
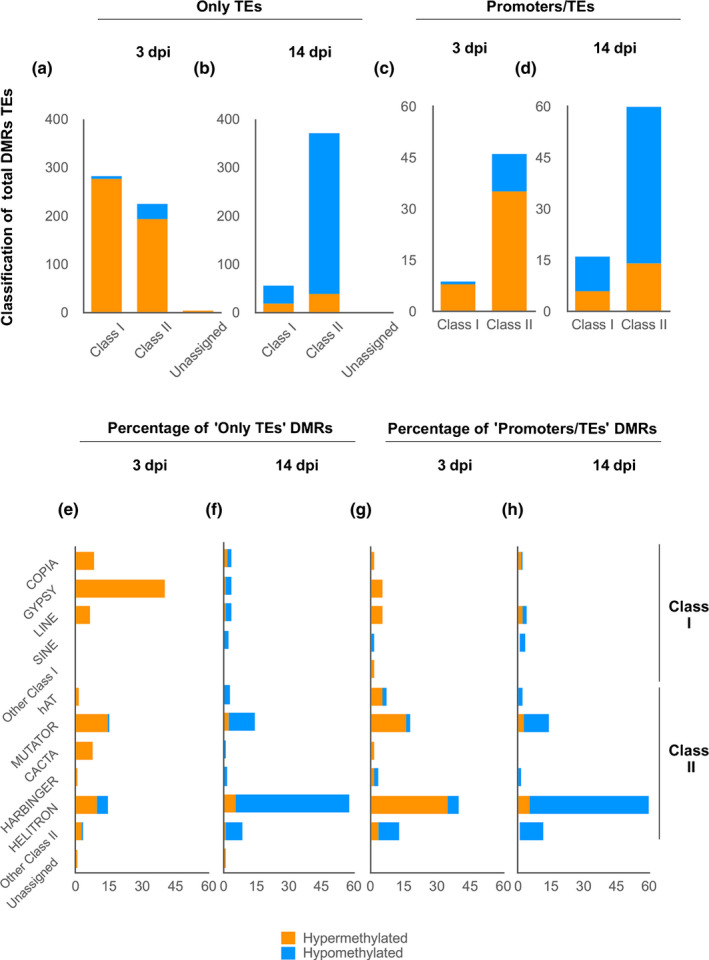
Classification of differentially methylated regions (DMRs; methylation difference > 15%) overlapping transposable elements (TEs). Differentially methylated regions overlapping TEs were classified into those that only overlapped TEs (‘Only TEs’) and those that also overlapped promoters (‘Promoters/TEs’). (a–d) Percentages of DMRs overlapping Only TEs (a, b) and Promoters/TEs (c, d) that map to class I (retrotransposons) and class II (DNA TEs) TEs, at 3 d post‐infection (dpi) (a, c) and 14 dpi (b, d). (e–h) Percentages of DMRs overlapping Only TEs (e, f) and Promoters/TEs (g, h) that map to different transposon superfamilies of class I (COPIA, GYPSY, LINE and SINE; Slotkin & Martienssen, 2007; Buisine *et al*., 2008) and class II (MUTATOR, EnSPm/CACTA, hAT, PIF‐HARBINGER, POGO, Tc‐MARINER and HELITRON; Underwood *et al*., 2017), at 3 dpi (e, g) and 14 dpi (f, h). In all cases, hypermethylation is shown in orange and hypomethylation in blue. Transposons not included in these superfamilies were categorized as ‘other class I’, ‘other class II’ or ‘unassigned’. The inclusion of RathE1_cons and RathE2_cons as a retrotransposon was considered following the work of Buisine *et al*. (2008).

We further classified DMRs overlapping TEs (‘Only TEs’) and those overlapping TEs and promoters (‘Promoters/TEs’), as well as those overlapping two different TE types: class I (retrotransposons that function via intermediate RNAs and reverse transcriptase) and class II (DNA‐TEs that function via a DNA intermediate and transposase). Interestingly, at 3 dpi, DMRs in ‘Only TEs’ were predominantly hypermethylated retrotransposons (Fig. 4a) in the GYPSY superfamily (Fig. 4e). By contrast, the predominant DMRs in ‘Promoters/TEs’ were DNA transposons (Fig. 4c) in the HELITRON and MUTATOR superfamilies (Fig. 4g). Furthermore, the proportion of DMRs matching retrotransposons (55.6%) is much higher than the proportion of retrotransposons (25.4%) among the TEs identified within the Arabidopsis genome in TAIR (Table S6). Similarly, the GYPSY superfamily represents 13.4% of TEs within the genome but 40% of TEs in DMRs at 3 dpi (Table S6), clearly indicating preferential changes in DNA methylation at 3 dpi (Fig. 4). At 14 dpi, the pattern is different (preferential towards DNA transposons vs retrotransposons at 3 dpi, in HELITRON and MUTATOR; Fig. 4b,d,f,h) and 86% of TEs overlap with DNA transposons, compared to 74.5% in the Arabidopsis genome (Table S6). All these data point to a dynamic and contrasting DNA methylation landscape during infection.

### Functional analysis of the main Arabidopsis DNA methylases and demethylase ROS1 confirm the relevance of methylation dynamics during *Meloidogyne javanica* infection

**Fig. 5:**
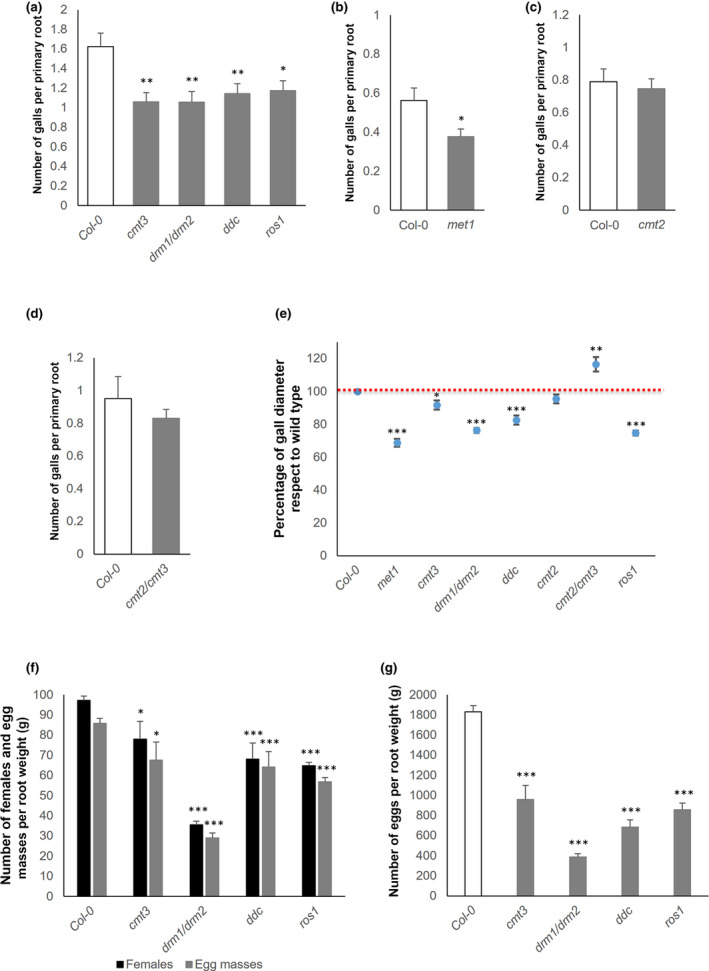
Arabidopsis mutants impaired in methyltransferases and DNA demethylase ROS1 are more resistant to root‐knot nematode infection than control plants. (a–d) Infection tests with *Meloidogyne javanica* showing the number of galls formed in mutant lines (*cmt3*, *drm1/drm2*, *ddc*, *ros1*, *met1*, *cmt2* and *cmt2/3*) per primary root following Olmo *et al*. (2017) compared with the control Col‐0 plants (*n* ≥ 111 plants/line). (e) Gall diameter (blue dots; *n* ≥ 25 galls/line) at 14 d post‐infection relative to that of Col‐0 (the red dotted line indicates the 100% value on the *y*‐axis). (f, g) Nematode reproduction parameters in the mutants relative to Col‐0 in soil (*n* ≥ 7), number of females and number of egg masses per root weight (in grams; f) and total number of eggs per root weight (in grams; g). Asterisks represent significant differences from Col‐0 control values according to Student’s *t*‐test (*, *P* < 0.05; **, *P* < 0.01; ***, *P* < 0.001). Values are means ± SE.

To confirm a functional role of DNA methylation/demethylation in galls, we performed infection tests in Arabidopsis mutant lines in which methyltransferase and DNA demethylase ROS1 activities were compromised. Infection was performed according to Olmo *et al*. (2017). We found a significant decrease in the infection rates in the DNA methyltransferase mutants (*cmt3*, *drm1/drm2*, *ddc* and *met1*) and in the DNA demethylase *ros1* mutant compared to the Col‐0 wild‐type control (*P* < 0.05; Fig. 5a,b). By contrast, no significant differences were encountered for *cmt2* or *cmt2/cmt3* (Fig. 5c,d). Additionally, the gall diameter in all DNA methyltransferase mutants (except *cmt2* and *cmt2/cmt3*) and ROS1‐glycosylase was significantly smaller than in galls formed in Col‐0 (*P* < 0.05; Fig. 5e). The most noticeable reduction was found in the *met1*‐*3* mutant (147 ± 5.0 μm), followed by *ros1*, as compared with Col‐0 (215 ± 4.9 μm; Fig. 5e).

**Fig. 6:**
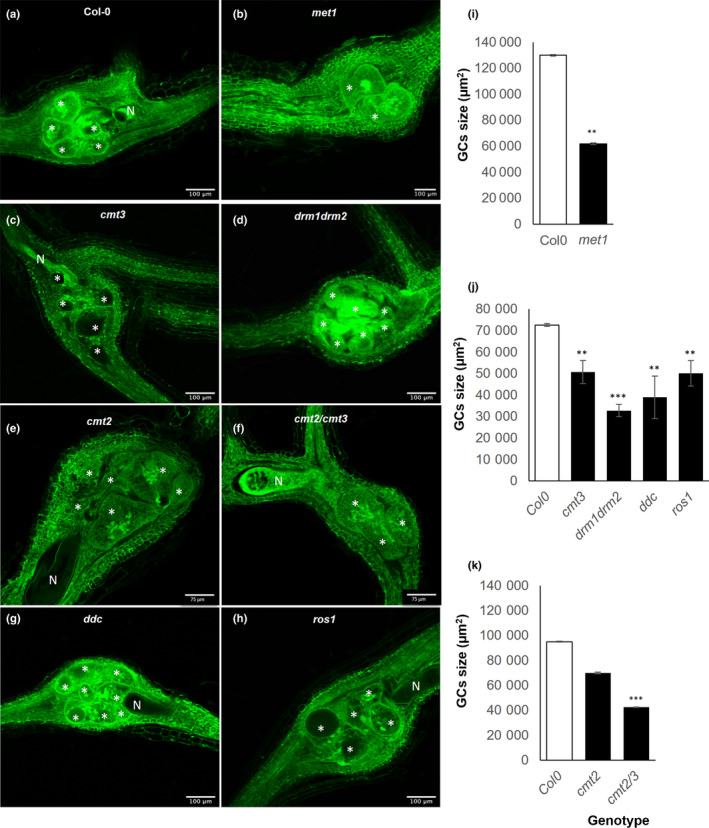
Arabidopsis mutants impaired in methyltransferases and DNA demethylase ROS1 showed altered feeding site development compared to control plants. (a–h) Confocal micrographs of galls formed by *Meloidogyne javanica* in different methylase mutants: *met1* (b), *cmt3* (c), *drm1/drm2* (d), *cmt2* (e), *cmt2/3* (f), *ddc* (g) and *ros1* (h), as compared to Col‐0 (a). (i–k) The average size of the giant cells (GCs) within a gall in *met1* and Col‐0 (i), *cmt3*, *drm1/drm2*, *ddc*, *ros1* and Col‐0 (j) and *cmt2*, *cmt2/3* and Col‐0 (k) (*n* ≥ 4 galls per line; 5 sections per gall). Asterisks represent significant differences in the GC sizes (μm^2^) of *met1*, *cmt3*, *cmt2/3*, *drm1/drm2*, *ddc* and *ros1* compared to Col‐0, according to Student’s *t*‐test (**, *P* < 0.01; ***, *P* < 0.001). Values are means ± SE. Bars: (a–d, g, h) 100 μm; (e, f) 75 μm. N, nematode. GCs are labelled with a white asterisk. Confocal microscopy was performed using a Leica TCS SP8 laser scanning confocal microscope (Leica, Wetzlar, Germany).

These results are also consistent with the infection and reproduction parameters for soil‐grown plants, in which all mutants tested showed significant differences (Fig. 5f,g). Hence, reproductive parameters also confirmed the impact of these genes on the ability of nematodes to complete their life cycle (Fig. 5f,g). Interestingly, GCs in all mutants were smaller compared to Col‐0, except for *cmt2* (Fig. 6a–k; Video S1–S8), with marked differences particularly evident in *met1* (*P* < 0.01; Fig. 6b,i; Video S7) and *drm1/drm2* (Fig. 6d,j; Video S8). These results, together with the evidence presented in the previous sections, highlight the crucial role not only of the *de novo* DRM2/1 methylation mediated by sRNAs (RdDM) and direct methylation maintenance (MET1, CMT3), but also of the demethylation mediated by ROS1 activity during *M. javanica* infection, in line with the DNA methylation dynamics observed at early and late infection stages (Figs 1, 2, 3, 4).

### DNA methylation within the early galls is predominant in the giant cells

Galls are pseudo‐organs formed by diverse cell types including GCs, and, in Arabidopsis and *S*. *lycopersicum*, gene expression of the isolated GCs is characterized by widespread gene repression (Barcala *et al*., 2010; Portillo *et al*., 2013). In order to focus on the epigenetic changes that correlate with the timing of general gene repression in GCs (Barcala *et al*., 2010) and may contribute to early gall/GC development, we focused on the 3 dpi stage for further analyses.

**Fig. 7:**
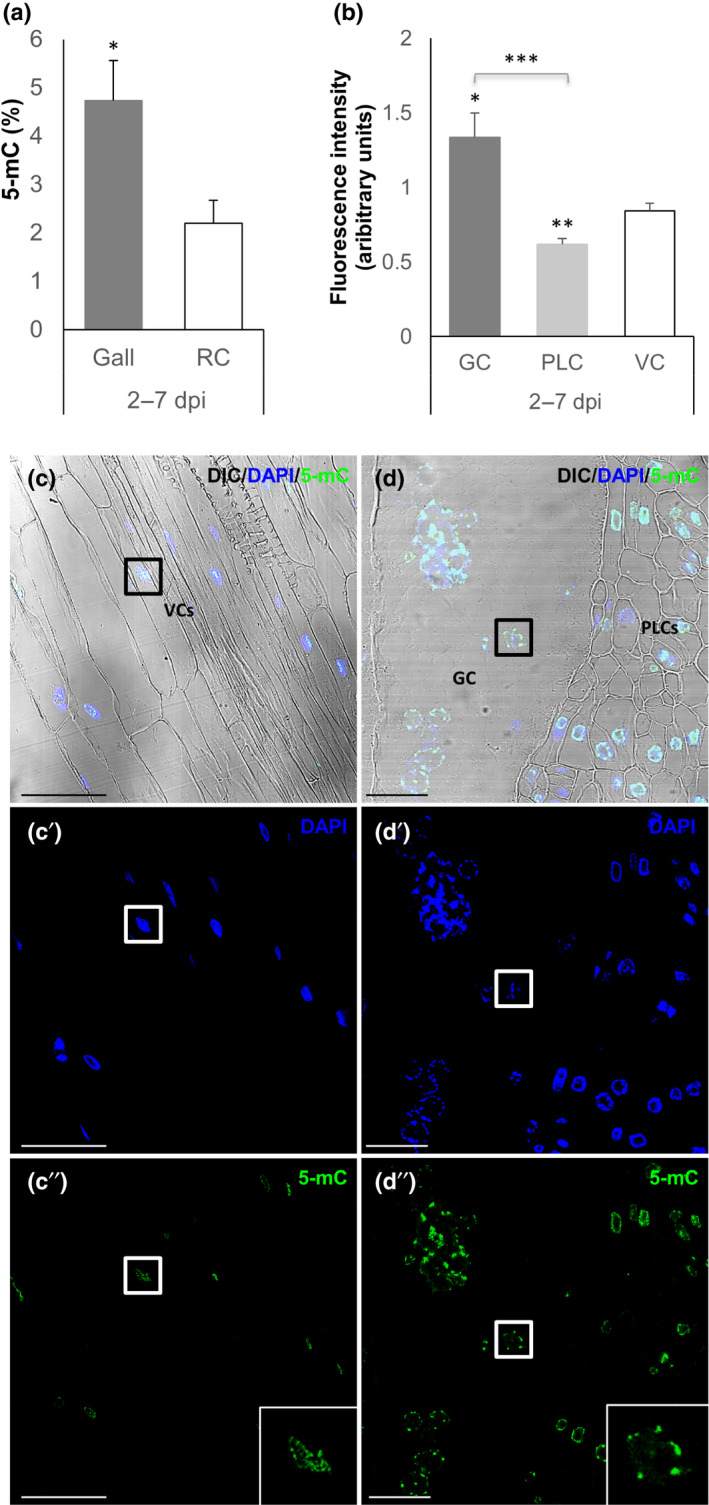
DNA methylation in *Solanum lycopersicum* galls and giant cells (GCs) at early stages of infection (2–7 d post‐infection; dpi). (a) Global 5‐methylcytosine (5‐mC) DNA methylation (in percentage) in galls and uninfected control root segments (RCs), quantified using an ELISA‐based kit (see the Materials and Methods section; ‘Quantification of global 5‐methylcytosine’ sub‐section). (b) Quantification of the 5‐mC immunofluorescence signal calculated for the nuclei of GCs, the proliferative cells (PLCs) formed within the gall and the vascular cylinder cells (VCs) of the RCs. In the histogram, immunofluorescence intensity is shown in arbitrary units, calculated from confocal microscopy maximum projection images (see the Materials and Methods section; ‘Immunofluorescence and confocal microscopy’ sub‐section). Values are means ± SE (‘n’ indicates number of nuclei; GCs, *n* = 43; PLCs, *n* = 57; VCs, *n* = 78). (c–c′′, d–d′′) Representative micrographs of 5‐mC immunofluorescence in control vascular cylinder cells (c, c′ and c′′) and infected roots (d, d′ and d′′); Nomarski's differential interference contrast (DIC) images show the cellular organization of the different tissues (c, d). Confocal micrographs for 4′,6‐diamidino‐2‐phenylindole (DAPI) staining (blue) (c′, d′) and the 5‐mC immunofluorescence signal from the nuclei (green) (c′′, d′′). A giant cell nucleus (right) and vascular cell nucleus (left) are highlighted in each image. In (c) and (d), VCs, PLCs and a GC are also indicated. Bars: (c–c′′) 50 μm; (d–d′′) 25 μm. Asterisks in (a) and (b) indicate significant differences according to Student’s *t*‐test ((a); *n* = 8) or the Kruskal–Wallis nonparametric test followed by Dunn's post hoc test (b) (*, *P* < 0.05; **, *P* < 0.01 and ***, *P* < 0.001). Confocal microscopy was performed using a Leica TCS‐SP5‐AOBS.

To determine the relative contributions of different cell types within galls (i.e. the GCs and surrounding cells) to the global hypermethylation pattern observed in 3 dpi galls, we exploited the technical tractability of tomato (morphology preservation is poor in early galls in Arabidopsis). We chose to analyze the spatial distribution of DNA methylation using immunolocalization of 5‐mC in early tomato galls as they also showed significant global hypermethylation compared to uninfected roots (Fig. 7a). The corrected immunofluorescence signal (see the Materials and Methods section; ‘Immunofluorescence and confocal microscopy’ sub‐section) emitted by GCs, the proliferative cells surrounding the GCs (PLCs), and the nuclei of vascular cylinder cells from the uninfected root segments (VCs) were compared. Significant differences were observed between GCs and VCs, GCs and PLCs, and between PLCs and VCs nuclei (Fig. 7b), and these differences can be distinguished in the representative images shown in Fig. 7(c) (compare panel (c) with (d) and (c′′) with (b′′)). The higher degree of fluorescence observed in GCs compared to PLCs and VCs suggests a predominant contribution of GCs to the general hypermethylation described by ELISA in tomato galls (Fig. 7a). Taken together, both the timing (Fig. 1) and location (Fig. 7), of DNA methylation in galls is consistent with the downregulation of gene expression in early developing GCs.

### *Meloidogyne javanica* induces chromosome region methylation patterns matching sRNA distribution during early infection

**Fig. 8:**
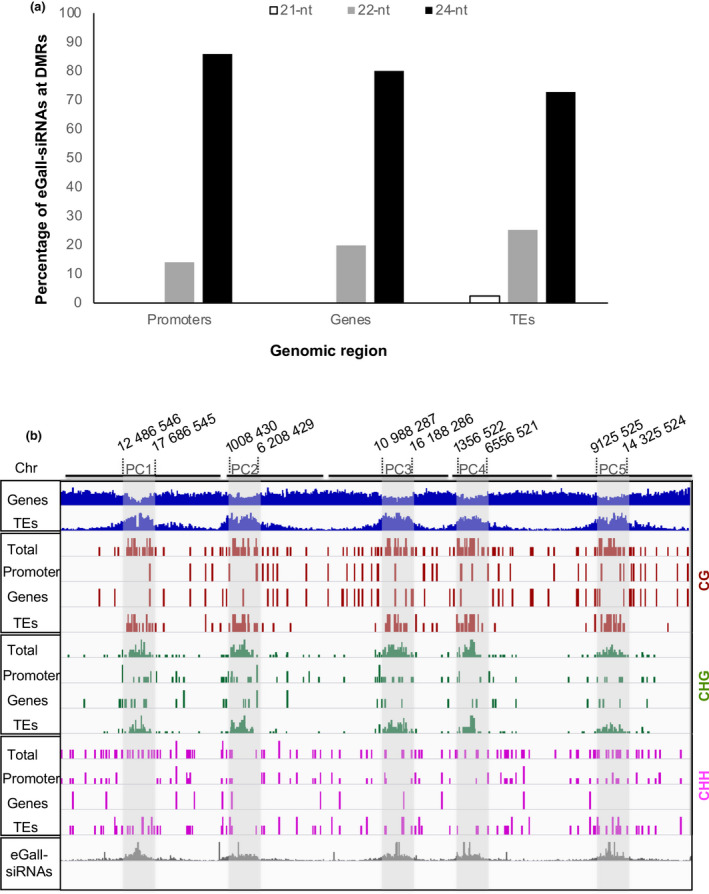
Distribution of differentially methylated regions (DMRs; methylation difference > 15%) and siRNAs overlapping promoters, genes and transposable elements (TEs) along the Arabidopsis chromosomes. (a) Classification of siRNAs exclusive to galls and not present in control roots (eGall‐siRNAs) that match DMRs overlapping promoters, genes and TEs according to nucleotide (nt) length: white, 21‐nt; grey, 22‐nt; black, 24‐nt. (b) The Arabidopsis genome is shown as a continuum of the five chromosomes (Chr). Arabidopsis chromosomes are classified into three regions: centromere (CEN), pericentromeric region (PC) and chromosome arm regions (AR). Pericentromeric regions are defined as regions in which the gene coverage in 1 Mb is ≤ 40%. The DMRs matching genes and TEs according to TAIR10 are shown in blue, and the pericentromeric regions (PCs) are highlighted with light grey bands for the five Arabidopsis chromosomes. The DMRs overlapping promoters, genes and TEs identified in each methylation context are coloured as follows: brown, CG; green, CHG; magenta, CHH. The differentially accumulated eGall‐siRNAs (*P* < 0.05) are shown in grey at the bottom of the figure.

To analyze whether sRNAs may drive the hypermethylation found in early galls, we compared the DMR data with available sRNA‐seq data for equivalent biological samples (3 dpi Arabidopsis‐*M. javanica* galls; Cabrera *et al*., 2016). Interestingly, most of the siRNAs that are exclusively present in galls and not in control roots (eGall‐siRNAs) overlapped with DMRs matching TEs, followed by those targeting DMRs in promoters and genes (Table S4). This indicates a correlation between the differential accumulation of siRNA in galls, particularly those that are gall‐distinctive (eGall; siRNAs only present in galls), and gall DMRs. The predominant eGall‐siRNA size in TEs, promoters and genes was 24‐nt, followed by 22‐nt (Fig. 8a; Table S4), that are usually products of DCL3 and DCL2, respectively. Neither promoters nor genes seem to be regulated by DCL4‐dependent 21‐nt siRNAs (Table S4; Fig. 8a).

Interestingly, the distribution pattern of eGall‐siRNAs matched the methylation distribution in the CHG and CG contexts along the five chromosomes, was concentrated at PC regions, and correlated with the predominant retrotransposon locations in the Arabidopsis genome (Fig. 8b; Simon *et al*., 2015), also matching the DMR distribution in retrotransposons at 3 dpi (Figs 2, 3, 4). However, those DMRs methylated in the CHH context extend along the chromosomes (Fig. 8b) and match the DMR distribution at promoters in the ARs (Figs 2, 3, 4). Taken together, these data indicate that the accumulation of eGall‐siRNAs in retrotransposons could be related to their DNA hypermethylation profiles, and is clearly in agreement with the finding that several retrotransposon superfamilies which are well‐known targets of the RdDM pathways (i.e. ATCOPIA48 and ATHILA2; Zhang *et al*., 2006), are downregulated at 3 dpi in Arabidopsis galls (Ruiz‐Ferrer *et al*., 2018; Fig. S1a). In addition, in galls at 14 dpi the expression of both retrotransposons significantly increased respect to galls at 3 dpi (Fig. S1b), indicating a dynamic in the repression/activation of major families of retrotransposons. Furthermore, differences in expression between the 3 dpi galls and uninfected tissue were not significant in the *dmr1/2* mutant background (Fig. S1c).

### Impact of gall and giant cell DNA methylation on gene expression at early infection stages

We performed RNA‐seq on 3 dpi galls (Table S3) from the same biological samples as those used for MethylC‐seq to ensure consistent comparisons between methylation patterns and gall transcriptomics, increasing the chances of finding reliable correlations (Silva *et al*., 2019).

The reliability of our RNA‐seq analysis was validated as it was consistent with the findings of a previous analysis of a gall microarray at the same infection stage (3 dpi; Barcala *et al*., 2010) that is, 84.5% of the DEGs in the 3‐dpi gall microarray were common with the RNA‐seq analysis (Fig. S2a). Additionally, a comparison with the DEGs from micro‐dissected GCs (3 dpi; Barcala *et al*., 2010) showed that around half (48.3%) of the total DEGs in GCs are common to both analyses (Fig. S2b), indicating that a high proportion of transcripts from GCs in the total population of gall RNAs were detected by RNA‐seq. The fact that 46% of the characteristic transcripts that peak in different cell‐cycle stages (Menges *et al*., 2002) are DEGs in galls at 3 dpi provides further evidence of the large contribution of the early GC transcripts to the gall RNA‐seq transcriptome. The largest proportion of upregulated genes were characteristic of M phase, followed by G1 phase. Consequently, M phase upregulated transcripts were overrepresented (*χ* ^2^ test with *P* < 0.05), in accordance with the active cell divisions taking place in the early stages of GC differentiation (Table S7).

Differentially expressed genes were classified into categories based on gene ontology using mapman (Usadel *et al*., 2009; Table S8). The category ‘microRNA, natural antisense, etc’ was overrepresented (Fig. S2c; Table S9), and most of these DEGs correspond to natural antisense transcripts. In line with this, 15 out of 16 key genes involved in Arabidopsis methylation pathways (Matzke & Mosher, 2014; Cuerda‐Gil & Slotkin, 2016) were upregulated in the RNA‐seq analysis (e.g. METHYLTRANSFERASE 1 (MET1), CHROMOMETHYLASE 2 and 3 (CMT2 and CMT3, respectively) and DOMAINS REARRANGED METHYLASE 1 (DRM1; Fig. S3)).

To analyze the putative impact of selective gall DNA methylation in gene expression, we identified the gall DEGs and GC DEGs (Barcala *et al*., 2010) within the genes and promoters that overlapped with DMRs (minimum methylation difference 15%; Table S5). Parameters for the cutoff of DEGs in the RNA‐seq were set up at an adjusted *P*‐value < 0.05 (Table S3b) to mimic as much as possible the criteria used in the GC microarray. Of those DMRs overlapping with a gene (102 DMRs; 99 different genes), 23 were DEGs, most of which were differentially methylated in the CG context and hypermethylated (Tables 1, S10). Of these 23 DEGs, 26.1% mapped siRNAs, with a high abundance of eGall‐siRNAs (83.3%), which were either 24‐nt or 22‐nt in length, relative to those that are distinctive of control roots (16.7%; 1; Table S4).

Among the DMRs overlapping gene promoters (100 DMRs; 100 different genes), 33 corresponded to DEGs (Table 2). They were found to be preferentially methylated in the CHH context and were hypermethylated (Tables 2, S10). Interestingly, 19 out of the 33 DMRs matched transposons, and most were DNA‐TEs (class II transposons). Of these 33 DEGs, 24.4% were targeted by siRNAs, with a higher abundance of eGall‐siRNAs (50%) and with equal distributions of those that are 24‐nt and 22‐nt in length (Table S4).

Gene ontology enrichment analysis of the genes in the correlation list (Tables 1, 2), showed that ‘RNA’ and ‘Protein’ were the predominant categories (Fig. S4). Interestingly, in the ‘RNA’ category, the vast majority of the genes encoded transcription factors. Furthermore, there were several genes encoding proteins with basic cellular functions described in other biological systems, such as CHR19, a chromatin remodeler, the replication protein A (RPA2B), and a histone deacetylase (HDT2), among others (Tables 1, 2). Four DEGs in GCs that were not common to the gall RNA‐seq results, also matched DMRs; three were hypermethylated and downregulated (Table 3).

**Table 1:** Genes overlapping differentially methylated regions (DMRs; methylation difference > 15%) and differentially expressed genes (DEGs) in Arabidopsis galls induced by *Meloidogyne javanica* at 3 d post‐infection.

Genomic region	Gene ID	Description	Methylation context	Difference of methylation	Log_2_FC	TE in the same bin	TE Superfamily	Log_2_ GCs
Gene	AT1G66340	ETHYLENE RESPONSE 1 (ETR1)	**CG**	**15**	**−0.32**			
	AT1G73390	Endosomal targeting BRO1‐like domain‐containing protein	**CG**	**36**	**−0.4**			**−2.09**
	AT2G02090	CHROMATIN REMODELING 19 (CHR19)	**CG**	**23**	**−0.42**			
	AT2G20980	MINICHROMOSOME MAINTENANCE 10 (MCM10)	**CG**	**21**	**1.05**			
	AT3G02920	REPLICATION PROTEIN A 2B, RPA2B	**CG**	**−25**	**1.13**			
	AT3G11130	CLATHRIN HEAVY CHAIN 1 (CHC1)	**CG**	**−29**	**−0.32**			
	AT3G20640	Basic helix‐loop‐helix (bHLH) DNA‐binding superfamily protein	**CG**	**−100**	**0.6**			
	AT3G24518	Natural antisense transcript overlaps with AT3G24520	**CG**	**25**	**−1.51**			
	AT3G27610	Nucleotidylyl transferase superfamily protein	**CG**	**−24**	**−0.41**			
	AT3G43600	ALDEHYDE OXIDASE 2 (AAO2)	**CG**	**31**	**0.3**			
	AT3G45240	GEMINIVIRUS REP INTERACTING KINASE 1 (GRIK1)	**CG**	**37**	**0.31**			
	AT3G45840	Cysteine/Histidine‐rich C1 domain family protein	**CG**	**20**	**−1.28**			
	AT3G60415	Phosphoglycerate mutase family protein	CHH	**19**	**5.12**			
	AT4G08400	EXTENSIN 7 (EXT7)	CHG	**16**	**3.22**			
	AT4G14760	Kinase interacting (KIP1‐like) family protein (NETWORKED 1B, NET1B)	**CG**	**24**	**0.53**			
	AT4G29305	LOW‐MOLECULAR‐WEIGHT CYSTEINE‐RICH 25 (LCR25)	CHH	**15**	**7.46**			
	AT4G32410	CELLULOSE SYNTHASE 1 (CESA1)	**CG**	**25**	**−0.34**			
	AT5G02400	POL‐LIKE 2 (PLL2)	**CG**	**17**	**−0.47**			
	AT5G06195	Novel transcribed region	**CG**	**36**	**1.74**			
	AT5G23120	HIGH CHLOROPHYLL FLUORESCENCE 136 (HCF136)	CHG	**−22**	**−1.24**			
	AT5G24670	EMBRYO DEFECTIVE 2820 (EMB2820)	CHH	**−22**	**−0.65**	AT5TE30560	HARBINGER	
	AT5G46290	3‐KETOACYL‐ACYL CARRIER PROTEIN SYNTHASE I (KASI)	**CG**	**20**	**0.41**			**−1.36**
	AT5G62190	DEAD/DEAH box RNA helicase (PRH75)	**CG**	**−16**	**−0.61**			

The methylation context more abundant in the DMRs (CG) is highlighted. The context and difference in methylation and corresponding gene expression (log_2_FC) are indicated, as well as the transposable elements (TEs) and their superfamilies present in the same bin, ID and gene description. Blue indicates DEGs that were found to be ‘GC distinctive’ when compared with the gall DEGs by Barcala *et al*. (2010). Red represents induced (and hypermethylated, compared to uninfected control roots) genes and green represents repressed (and hypomethylated) genes.

**Table 2:** Gene promoters overlapping differentially methylated regions (DMRs; methylation difference > 15%) and differentially expressed genes (DEGs) in Arabidopsis galls induced by *Meloidogyne javanica* at 3 d post‐infection.

Genomic region	Gene ID	Description	Methylation context	Difference of methylation	Log_2_FC	TE in the same bin	TE Superfamily	Log_2_ GCs
Promoter	AT1G27000	GRIP/coiled‐coil protein, putative (DUF1664)	**CHH**	**16**	**−0.27**			
	AT1G30490	PHAVOLUTA (ATHB9)	**CHH**	**21**	**0.6**			
	AT1G44970	PEROXIDASE9 (PRX9)	CG	**20**	**4.13**			
	AT1G51405	Myosin‐like protein	**CHH**	**22**	**1.61**	AT1TE63150	HELITRON	
	AT1G66340	ETHYLENE RESPONSE 1 (ETR1)	**CHH**	**−21**	**−0.32**	AT1TE81245	OTHER CLASS II	
	AT1G66345	MITOCHONDRIAL INTRON SPLICING FACTOR 26 (MISF26)	CG	**15**	**0.56**			
	AT2G01680	Ankyrin repeat family protein	**CHH**	**−19**	**−0.3**	AT2TE01275	SINE	
	AT2G03980	GDSL‐motif esterase/acyltransferase/lipase	**CHH**	**18**	**1.07**	AT2TE05710	HARBINGER	
	AT2G26135	RING FINGER ABA‐RELATED 9 (RFA9)	**CHH**	**22**	**4.71**	AT2TE48010	hAT	
	AT2G31070	TCP DOMAIN PROTEIN 10 (TCP10)	**CHH**	**21**	**−1.72**			
	AT2G39100	RING/U‐box superfamily protein	CG	**−23**	**−0.62**			
	AT2G43290	MULTICOPY SUPPRESSORS OF SNF4 DEFICIENCY IN YEAST 3 (MSS3)	**CHH**	**16**	**1.01**	AT2TE81445	HELITRON	
	AT3G04870	ZETA‐CAROTENE DESATURASE (ZDS)	**CHH**	**17**	**−0.35**	AT3TE05640	HELITRON	
	AT3G22930	CALMODULIN‐LIKE 11 (CML11)	**CHH**	**35**	**2.26**	AT3TE34090	MUTATOR	
	AT3G26210	CYTOCHROME P450, FAMILY 71, SUBFAMILY B, POLYPEPTIDE 23 (CYP71B23)	CHG	**25**	**−0.86**			
	AT3G27030	Transmembrane protein	CG	**16**	**−0.93**			
	AT3G28130	Nodulin MtN21‐like transporter family protein (UMAMIT44)	CHG	**16**	**0.79**	AT3TE43470	MUTATOR	
	AT3G29185	BIOGENESIS FACTOR REQUIRED FOR ATP SYNTHASE 1 (BFA1)	CHG	**28**	**−0.42**	AT3TE46395	LINE	
	AT3G29810	COBRA‐LIKE PROTEIN 2 PRECURSOR (COBL2)	**CHH**	**15**	**1.3**	AT3TE48840	HELITRON	
	AT3G45160	Putative membrane lipoprotein	**CHH**	**22**	**1.22**	AT3TE67015	HELITRON	**−1.72**
	AT3G55310	NAD(P)‐binding Rossmann‐fold superfamily protein	CG	**29**	**−1.97**	AT3TE83365	COPIA	
	AT4G00755	F‐box family protein	**CHH**	**−27**	**−0.88**	AT4TE01700	HELITRON	
	AT4G13110	BSD domain‐containing protein	CG	**18**	**−0.59**			
	AT4G13160	Zein‐binding protein (Protein of unknown function, DUF593)	**CHH**	**20**	**−0.49**	AT4TE33480	HELITRON	
	AT4G25020	D111/G‐patch domain‐containing protein	**CHH**	**−48**	**2.24**			
	AT5G05250	Hypothetical protein	CG	**31**	**−1.09**			
	AT5G06195	Novel transcribed region	CG	**36**	**1.74**			
	AT5G17870	PLASTID‐SPECIFIC 50S RIBOSOMAL PROTEIN 6 (PSRP6)	**CHH**	**16**	**−0.34**			
	AT5G22650	HISTONE DEACETYLASE 2B (HD2B; HDT2)	CG	**27**	**0.43**	AT5TE27225	MUTATOR	
	AT5G24670	EMBRYO DEFECTIVE 2820 (EMB2820)	**CHH**	**−22**	**−0.65**	AT5TE30560	HARBINGER	
	AT5G30495	Fcf2 pre‐rRNA processing protein	**CHH**	**16**	**0.56**	AT5TE41200	HELITRON	
	AT5G46290	3‐KETOACYL‐ACYL CARRIER PROTEIN SYNTHASE I (KASI)	CG	**20**	**0.41**			**−1.36**
	AT5G53060	REGULATOR OF CBF GENE EXPRESSION 3 (RCF3)	**CHH**	**−18**	**0.48**			

The methylation context more abundant in the DMRs (CHH) is highlighted. The context and difference in methylation and corresponding gene expression (log_2_FC) are indicated, as well as the transposable elements (TEs) and their superfamilies present in the same bin, ID and gene description. Blue indicates DEGs that were found to be ‘GC distinctive’ when compared with the gall DEGs by Barcala *et al*. (2010). Red represents induced (and hypermethylated, compared to uninfected control roots) genes and green represents repressed (and hypomethylated) genes.

**Table 3:** Differentially expressed genes (DEGs) of giant cells (GCs; Barcala *et al*., 2010) overlapping differentially methylated regions (DMRs; methylation difference > 15%) described in this study.

Genomic region	Gene ID	Description	Methylation context	Difference of methylation	Log_2_ value for GCs	TE in the same bin	TE Superfamily
Gene	**AT2G05755**	Nucleotide/sugar transporter family protein	**CHH**	**18**	**−2.61**	AT2TE09990	HELITRON
	**AT2G46130**	WRKY DNA‐BINDING PROTEIN 43 (WRKY43)	**CHH**	**−25**	**−0.99**	AT2TE86210	OTHER CLASS II
Promoter	**AT3G45680**	Major facilitator superfamily protein	**CHG**	**25**	**−2.15**	AT3TE67950	SINE
	**AT5G40500**	Hypothetical protein	**CG**	**29**	**−1.35**		

The context and difference in methylation and corresponding gene expression (log_2_FC) are indicated, as well as the transposable elements (TEs) and their superfamilies present in the same bin, ID and gene description. Red represents induced (and hypermethylated, compared to uninfected control roots) genes and green represents repressed (and hypomethylated) genes.

## Discussion

Transcriptomic analyses of Arabidopsis infected with *Meloidogyne* spp. indicated a very high degree of gene repression at early stages of infection, particularly in GCs, that is conserved in tomato (Barcala *et al*., 2010; Portillo *et al*., 2013). These results are in agreement with the finding that, several microRNAs that act by repressing plant genes encoding transcription factors were induced in galls (i.e. miR390/*ARF3*, miR159/*MYB33* and miR172/*TOE1*) at different stages of the RKN infection (Cabrera *et al*., 2016; Medina *et al*., 2017, Díaz‐Manzano *et al*., 2018; respectively). Additionally, retrotransposons from several superfamilies were dramatically repressed in early galls (Ruiz‐Ferrer *et al*., 2018). Moreover, the differential accumulation of sRNAs in early galls, such as rasiRNAs, as compared to control tissues strongly suggests the participation of RdDM pathways during nematode establishment (Cabrera *et al*., 2016; Ruiz‐Ferrer *et al*., 2018). However, no information is available regarding the changes in DNA methylation patterns, their correlation with changes in gene expression and sRNA accumulation, or their functional implications during gall/GC formation by RKNs in dicotyledonous species.

### The relevance of methylation dynamics during *Meloidogyne javanica* infection

*Meloidogyne javanica* induces generalized hypermethylation during early gall development in tomato and Arabidopsis, as assessed here using two independent methods (3 dpi; Figs 1, 7). Several studies reported methylation changes during pathogen attack in plants, such as bacterial infection (Dowen *et al*., 2012; Yu *et al*., 2013), fungal infection (Le *et al*., 2014; López Sánchez *et al*., 2016) and infection by syncytia‐forming nematodes, *Heterodera* spp. (reviewed in Hewezi, 2020). However, this is the first report of gall‐localized hypermethylation during a plant–nematode interaction. By contrast, studies in *Heterodera* spp. and in the RKN *M. graminicola* in monocotyledonous species (rice; Atighi *et al*., 2020) showed general hypomethylation at early and mid stages of infection. Differences in the evolutionary origins of sedentary endo‐parasitism, parasite life cycles, and experimental conditions could explain the observed divergences, or maybe different types of nematodes elicit divergent epigenomic strategies that may also depend on the host plant. Conversely, similarities in the transcriptomic profiles were observed between GCs and crown‐galls formed by *Agrobacterium tumefaciens* (Barcala *et al*., 2010), showing generalized hypermethylation (Gohlke *et al*., 2013) and demonstrating that there are parallels between the response to RKN infection and other plant–pathogen interactions. Together, these findings suggests that different epigenomic strategies are used by different pathogen species during the interaction with their hosts.

However, no global differences in DNA methylation were encountered in medium/late galls relative to the control roots (14 dpi; Fig. 1). The contrast between early and late infection stages suggests a dynamic DNA methylation landscape during the infection. Accordingly, the distribution of differentially methylated regions (DMRs) was markedly different between the infection stages (Figs 2, 3, 4). Most DMRs were hypermethylated at 3 dpi and overlapped TEs, mostly retrotransposons located at pericentromeric regions of the chromosomes with a predominance of the GYPSY superfamily (Figs 2, 3, 4). However, at 14 dpi, most DMRs were hypomethylated and overlapped DNA transposons located at the chromosome arms with a predominance of the HELITRON superfamily (Figs 2, 3, 4). Since there is no overall global change in the amount of 5‐mC‐methylation in 14 dpi galls compared to RCs, these findings strongly suggest active remodeling of the methylation landscape from early establishment to the medium/late stage of infection. Consequently, main methylase and demethylase functions were required for adequate gall and GC formation, as well as for nematode reproduction in Arabidopsis (Figs 5, 6). Thus, mutants of methylases involved in the RdDM pathway (DRM2) and methylation maintenance (CMT3, MET1), and a mutant of DNA demethylase ROS1, showed either reproduction and/or infection parameters, and gall size/GC size severely compromised, but this was not observed in CMT2 during early infection (Figs 5, 6). These results also highlight the crucial and dynamic role of direct maintenance of methylation in the CG (mainly MET1‐mediated), and CHG (mainly CMT3‐mediated) contexts (Pikaard & Scheid, 2014), but perhaps a limited role for CMT2 maintenance of CHH methylation at 3 dpi. In line with this, the decrease in the infection parameters in *drm1/drm2* with respect to the control line does not correspond to the reduction in the reproduction parameters (around 30% and 60–70%, respectively; Fig. 5). This may suggest that the relative contribution of different methylases/demethylases could change over the course of the infection. MET1 is specifically recruited to hemi‐methylated cytosines after DNA replication to methylate the newly synthesized unmethylated strand (Pikaard & Scheid, 2014), which should be essential during early GC formation due to an enhanced S phase as a consequence of repeated mitosis (de Almeida Engler & Gheysen, 2013), in accordance with the overrepresentation of M‐phase‐induced genes observed at 3 dpi in the RNA‐seq analysis (Table S7). The absence of a clear phenotype in *cmt2* galls reinforces the role of the DRM1/2 RdDM pathways during the early stages of *M. javanica* infection, not only in terms of CHG and CG methylation, but perhaps as a relevant mechanism of CHH methylation (Figs 5, 8; Pikaard & Scheid, 2014). This is further supported by the fact that genes encoding proteins involved in *de novo* methylation and maintenance, DNA‐methyltransferases (MET1, CMT2 and CMT3), ARGONAUTE 4 (AGO 4), DRM1, the RNA polymerase PolIV/PolV subunits (NRPD2/NRPE2, NRPD4/NRPE4) and INVOLVED IN DE NOVO 2 (IDN2), among others, were induced in galls at 3 dpi (Fig. S3).

Generalized hypermethylation, particularly in the CHH context, as observed in 3 dpi galls, has been described during somatic embryogenesis in soybean after auxin induction (Ji *et al*., 2019). In accordance with this result, in this study, the greatest differences in methylation between galls and control tissue were observed in the CHH context at 3 dpi (Fig. 1), consistent with previous studies that demonstrate an auxin maxima within early galls (Hutangura *et al*., 1999; Karczmarek *et al*., 2004; Absmanner *et al*., 2013; Cabrera *et al*., 2015; Kyndt *et al*., 2016; Olmo *et al*., 2020). Gall formation is a *de novo* post‐embryonic organogenesis process that shares characteristics with other developmental processes, such as lateral‐root initiation or callus formation, where auxins are the main orchestrating hormones (Olmo *et al*., 2020). The roles of various proteins involved in DNA methylation in the control of auxin biosynthesis and transport were recently described elsewhere (Forgione *et al*., 2019; Mateo‐Bonmatí *et al*., 2019). In this context, late‐developing galls (14 dpi) show low auxin responses (Karczmarek *et al*., 2004), in agreement with the lack of differences in the global methylation observed between galls and uninfected roots at this stage of infection (Fig. 1). Methylation changes observed in early galls might, therefore, be at least partially connected to enhanced auxin signaling pathways, although further research should be performed to clearly elucidate its role.

### DNA methylation within the early galls is predominant in the giant cells, matching sRNA distribution

We detected a selective hypermethylation in GCs using immunofluorescence targeted to 5‐mC, but not in the surrounding cells of the galls, which suggests that the methylation state of GCs makes a key contribution to that of the general gall hypermethylation at 3 dpi (Fig. 7). This constitutes a novel finding that might be related to the dramatic morphological and transcriptomic changes suffered by the plant in order to provide nourishing cells that support nematode development (Escobar *et al*., 2015). Some of these changes are reflected in the RNA‐seq analysis of galls at 3 dpi, where ‘GC‐distinctive’ transcripts defined in Barcala *et al*. (2010) are highly represented (Fig. S2) and M‐phase transcripts are overrepresented (Table S7), as expected from cells that undergo repeated mitosis with partial cytokinesis (reviewed in de Almeida Engler *et al*., 2015). Enhanced methylation due to endoreduplication within the nuclei at 2–7 dpi can be discarded, as mitosis is the predominant mechanism at early stages, but endoreduplication of nuclei starts later in the infection, a process that has only been directly analyzed in 40 dpi Arabidopsis galls (Vieira *et al*., 2014; de Souza *et al*., 2017).

Interestingly, at 3 dpi, 24‐nt siRNAs, the most abundant eGall‐siRNAs, followed by 22‐nt siRNAs, accumulated at DMRs overlapping TEs, promoters and genes, (Fig. 8); DCL3 and DCL2 can produce 24‐nt siRNAs and 22‐nt siRNAs, respectively. DCL3 mediates TGS (24‐nt siRNAs), but DCL2‐dependent siRNAs can mediate PTGS and TGS (22‐nt siRNAs), catalyzed by DRM2/DRM1 (Zheng *et al*., 2007; Naumann *et al*., 2011; Du *et al*., 2015; McCue *et al*., 2015). In accordance with these findings, the double mutant *drm1/2* was severely impaired in nematode infection and gall/GC development (Figs 5, 6). DRM2 catalyzes methylation in all three cytosine contexts (CG, CHG, CHH) in the RdDM pathway (Pikaard & Scheid, 2014). Three d post‐infection eGall‐siRNA accumulation matched DMRs and hypermethylation in the CHG context, followed by the GC context, mainly in TEs at pericentromeric chromosome regions correlating with retrotransposon locations; by contrast, CHH hypermethylation was less frequent, and expanded along the chromosomes, matching predominantly the distribution of DMRs at promoters, as well as DNA transposons (Figs 2, 4, 8; Ruiz‐Ferrer *et al*., 2018). Therefore, the severe phenotype of the galls of the double mutant *drm1/2* confirms the large contribution of the *de novo* RdDM methylation pathway during gall formation, presumably with a major role in CHG and CG *de novo* methylation in retrotransposons. These findings are also in agreement with observations of *Meloidogyne*‐resistant phenotypes resulting from mutations in crucial molecular components of the canonical and noncanonical RdDM pathways, such as DCL2, DCL3, DCL4 or RDR2, RDR6 (Ruiz‐Ferrer *et al*., 2018).

Furthermore, there is a robust correlation between the hypermethylation, the preferential accumulation of eGall‐siRNAs at retrotransposons (particularly of the GYPSY and COPIA superfamilies), and their repression in early galls (Figs 3, 4, 8, S1; Ruiz‐Ferrer *et al*., 2018). By contrast, in galls at 14 dpi the expression of retrotransposons ATCOPIA48 and ATHILA2 significantly increased with respect to galls at 3 dpi, indicating that the repression/activation of major families of retrotransposons is dynamic during the infection and correlates with the changes in the global methylation status of galls, as the general hypermethylation described at 3 dpi disappeared at 14 dpi (Fig. 1). Moreover, it also matched the high number of hypermethylated DMRs in the COPIA and GYPSY retrotransposon families at 3 dpi (Fig. 4e), and the low number of hypermethylated DMRs in the same retrotransposons at 14 dpi (Fig. 4b,f). These results strongly suggest that this could be a strategy in the early developing GCs for the stabilization of TEs. Furthermore, the repression of ATCOPIA48 and ATHILA2 observed in Col‐0 galls at 3 dpi was nearly abolished in the *drm1/2* mutant background (Fig. S1), suggesting the participation of those methylases in the regulation of retrotransposons in galls. Silencing and stabilization of transposons are typical of plant egg cells and embryos, with the aim of increasing genome integrity in the offspring (Feng *et al*., 2010). RdDM is also active during embryogenesis, with increased CHH methylation in the endosperm and young embryo (Chow *et al*., 2020). However, in Arabidopsis, TE silencing in the developing embryo is also assisted by endosperm‐derived 24‐nt sRNAs (Bouyer *et al*., 2017). Like GCs within the galls, rapidly differentiating cells from the columella within the root meristem exhibit hypermethylated TEs and an increased abundance of transcripts encoding RdDM pathway components (Fig. S3; Kawakatsu *et al*., 2016; Ruiz‐Ferrer *et al*., 2018). Thus, TE stabilization driven by RdDM could be relevant in the differentiation process that leads to gall/GC formation at early stages, where a reprogramming of gene expression occurs, while successive mitosis takes place predominantly in the GCs (de Almeida Engler & Gheysen, 2013; Table S7), consistent with the distinctive hypermethylation observed in GCs but not in the surrounding cells (Fig. 7). By contrast, in Arabidopsis plants infected with the cyst nematode *Heterodera shachtii*, a widespread hypomethylation was predominant and some hypermethylated regions were not clearly associated with siRNA abundance (Hewezi *et al*., 2017; Hewezi, 2020). Additionally, a majority of TEs, particularly DNA‐TEs, were induced, as observed under other environmental stresses (Piya *et al*., 2017). Therefore, the epigenomic changes observed in early‐galls seem to be more strongly related to post‐embryogenic developmental processes that require rapid cell proliferation and differentiation than to those typical of defense responses in other plant–pathogen interactions.

However, we should not forget the contribution of the PTGS pathway, which is consistent with the overrepresentation of the ‘microRNA, natural antisense’ DEG categories in the mapman data for galls at 3 dpi (Fig. S2) and with the defined role of several microRNAs during gall formation (i.e. miR172/TOE, miR390/ARF3, miR159/MYB33) (reviewed in Cabrera *et al*., 2018b; Jaubert‐Possamai *et al*., 2019). Thus, RKNs use several strategies at the transcriptional and post‐transcriptional level to reprogram their host plant cells.

Additionally, 52 DEGs identified in the transcriptomic analysis at 3 dpi correlated with DMRs overlapping genes and promoters, a low number compared to the total number of DEGs (Tables 1, 2), a tendency also described by other authors (Rambani *et al*., 2015; Hewezi *et al*., 2017), while in other *Meloidogyne*–monocotyledonous species studies no correlation was obtained at 3 dpi (Atighi *et al*., 2020). Most of those genes encode proteins that participate in critical basic cell functions that could be also crucial for gall/GC development, such as those regulating transcription or those involved in plant developmental processes or functions related to epigenetic pathways; for example, HISTONE DEACETYLASE 2B (HDT2) was shown to be upregulated and functional during callus formation from leaf explants (Lee *et al*., 2016; Tables 1, 2). HDTs interact with MET1 to silence transposons, regulating DNA methylation (Liu *et al*., 2012). Nevertheless, a direct relationship between DNA hypermethylation and repression, or *vice‐versa*, was not observed for all genes. Methylation at promoter regions is usually associated with gene silencing (To *et al*., 2015), but it can also be associated with gene activation as it could favor the binding of transcriptional activators or prevent the binding of transcriptional repressors (Zhang *et al*., 2018; Smith *et al*., 2020). Therefore, to assign the methylation state of a promoter/gene to its transcriptional pattern is a complex matter. Interestingly, most of the DEG promoters at 3 dpi were methylated in the CHH context, where DRM1/2‐mediated methylation seems to be the most prominent driving mechanism, and is probably mediated by the accumulated 24/22‐nt eGall‐siRNAs, as the contribution of CMT2 is quite limited, judging by the lack of phenotype in galls in mutant plants (Figs 5, 6, 8).

In conclusion, we have shown that galls formed by *M. javanica* in Arabidopsis and tomato are characterized by generalized hypermethylation at early stages, to which GCs are the main contributors with respect to other cell types within the gall. The lack of differences in global methylation between medium/late galls and RCs, as well as the remarkably different locations of DMRs between the two infection stages under study here, strongly suggest a dynamic remodeling of the epigenetic landscape in terms of DNA methylation during infection. Functional assays with Arabidopsis mutant lines point to the dynamic participation of methylases and demethylases during early–medium/late infection. Moreover, DNA methylation patterns and the accumulation of eGall‐siRNAs, as well as the retrotransposon repression described in early galls (3 dpi; Ruiz‐Ferrer *et al*., 2018), are likely regulated by DNA methylation and mediated by DRM2/1 via RdDM pathways and by maintenance methylases (CMT3, MET1). This process may contribute to the TE stability and therefore genome integrity that should be required for the dramatic reprogramming processes accompanying cell differentiation, which are concurrent with repeated mitosis during GC formation. Interestingly, most of the DEGs matching DMRs overlapping promoters or genes encode proteins that participate in critical basic cell functions which seem pertinent to GC and gall development. Further research with loss of function mutants for these genes will shed some light on their putative roles during gall formation.

## Author contributions

ACS and VR‐F performed most of the experiments, data collection, and *in silico* analysis of genomic and transcriptomic data, and participated in the interpretation of the results, as well as in the writing of the manuscript. CP, SYM and SEvdA performed and guided ACS in the *in silico* analysis of genomic and transcriptomic data. MFA, AM‐G, AG‐R and PA‐U performed some of the experiments. PST and EB participated in and guided some of the experiments. CF and SEvdA participated in the writing of the manuscript and interpretation of the results. CE planned, designed, and guided most of the research and participated in the interpretation of the results and in the writing of the manuscript. ACS and VR‐F contributed equally to this work.

## Supporting information


**Fig. S1** Expression of class I transposon elements (retrotransposons) and validation of MethylC‐sequencing (MethylC‐seq) for selected differentially methylated regions at 3 d post‐infection.
**Fig. S2** Differentially expressed genes in Arabidopsis galls/giant cells at 3 d post‐infection (dpi) with *Meloidogyne javanica*, according to RNA‐sequencing (RNA‐seq) and microarray analyses.
**Fig. S3** Expression levels of differentially expressed genes in galls at 3 d post‐infection (RNA‐seq) involved in several epigenetic processes.
**Fig. S4** Gene ontology of differentially expressed genes (RNA‐seq) overlapping differentially methylated regions in galls at 3 d post‐ infection.
**Methods S1** Detailed description of extended methods.

**Table S1** List of primers and Arabidopsis mutants used in this study.

**Table S2** Summary of general data from MethylC‐seq and processing, for galls induced by *Meloidogyne javanica* and controls at 3 d post‐infection (dpi) and 14 dpi.

**Table S3** Summary of general data from RNA‐seq and processing, for galls induced by *Meloidogyne javanica* and controls at 3 d post‐infection.

**Table S4** Percentages and numbers of unique siRNAs matching genes, promoters and transposable elements (TEs) identified in DMRs (methylation difference > 15%) in galls at 3 d post‐infection.

**Table S5** Table of general DMRs at 3 d post‐infection (dpi) and 14 dpi.

**Table S6** Percentage of TEs described in the Arabidopsis genome (TAIR10) and DMRs (methylation difference > 15%) matching TEs in Arabidopsis galls induced by *Meloidogyne javanica* (3 d post‐infection (dpi) and 14 dpi).

**Table S7** Total numbers and percentages of DEGs in galls formed by *Meloidogyne javanica* in Arabidopsis at 3 d post‐infection common with genes involved in different phases of the cell division cycle.

**Table S8** Differentially expressed genes classified according to categories represented in mapman.

**Table S9** Differentially expressed genes represented in the mapman category ‘microRNAs, natural antisense etc’, in galls at 3 d post‐infection.

**Table S10** Genes and promoters that overlapped a DMR (methylation difference > 15%) and were differentially expressed in galls at 3 d post‐infection (adjusted *P*‐value < 0.05).

**Video S1** Col0 gall at 14 dpi.

**Video S2** *ddc* gall at 14 dpi.

**Video S3** *cmt2* gall at 14 dpi.

**Video S4** *cmt3* gall at 14 dpi.

**Video S5** *cmt2‐cmt3* gall at 14 dpi.

**Video S6** *ros1* gall at 14 dpi.

**Video S7** *met1* gall at 14 dpi.

**Video S8** *drm1/2* gall at 14 dpi.
Please note: Wiley Blackwell are not responsible for the content or functionality of any Supporting Information supplied by the authors. Any queries (other than missing material) should be directed to the *New Phytologist* Central Office.

## Data Availability

Data openly available in a public repository that does not issue DOIs. The data that support the findings of this study are openly available in GEO at https://www.ncbi.nlm.nih.gov/geo/, under reference nos. GSE155171, GSE155853 and GSE156025.
