# Supplementary material for: The DNA methylation landscape of the root‐knot nematode‐induced pseudo‐organ, the gall, in Arabidopsis, is dynamic, contrasting over time, and critically important for successful parasitism
Source: New Phytol. 2022 Sep 2;236(5):1888–907. doi: 10.1111/nph.18395 (PMC9825882; doi:10.1111/nph.18395)
Supplement: Supplementary file 1 — Fig. S1 Expression of class I transposon elements (retrotransposons) and validation of MethylC‐sequencing (MethylC‐seq) for selected differentially methylated regions at 3 d post‐infection. Fig. S2 Differentially expressed genes in Arabidopsis galls/giant cells at 3 d post‐infection (dpi) with Meloidogyne javanica, according to RNA‐sequencing (RNA‐seq) and microarray analyses. Fig. S3 Expression levels of differentially expressed genes in galls at 3 d post‐infection (RNA‐seq) involved in several epigenetic processes. Fig. S4 Gene ontology of differentially expressed genes (RNA‐seq) overlapping differentially methylated regions in galls at 3 d post‐ infection. Methods S1 Detailed description of extended methods. [file NPH-236-1888-s019.pdf]

New Phytologist Supporting Information

Article title: The DNA-methylation landscape of the root-knot nematode-induced pseudo-organ, the gall, in Arabidopsis, is dynamic, contrasting over time, and critically important for successful parasitism

Authors: Ana Cláudia Silva, Virginia Ruiz-Ferrer, Sebastian Y. Müller, Clement Pellegrin, Patricia Abril, Ángela Martínez-Gómez, Almudena Gómez-Rojas, Eduardo Berenguer, Pilar S. Testillano, Maria Fe Andrés, Carmen Fenoll, Sebastian Eves-van den Akker and Carolina Escobar

Article acceptance date: 1 July 2022

Supplementary Figures

Figure S1 - Expression of Class I Transposon elements (retrotransposons) and validation of MethylC-seq on selected DMRs at 3 d post-infection (dpi; Methods S1). (a) Relative expression levels by quantitative polymerase chain reaction (qPCR) of ATHILA2 and ATCOPIA48, representatives of retrotransposon superfamilies in Col-0 galls *versus* Col-0 uninfected control roots, at 3 d post-infection (3 dpi), (b) in Col-0 galls at 14 dpi *versus* galls at 3 dpi and (c) in 3 dpi galls *versus* uninfected control roots from the *drm1/2* Arabidopsis background. Three independent biological replicates, with three technical replicates each were performed and normalized to Glyceraldehyde-3-Phosphate Dehydrogenase (GADPH; Ruiz-Ferrer *et al*., 2018). Differences from control values were significant at **P* < 0.05, ***P* < 0.01, and ****P* < 0.001 (two-tailed *t*-test). Values are means ± SE. (d) Validation of MethylC-seq on selected DMRs, at 3 dpi. All DMRs described in this table are classified by chromosome, start and end position on Arabidopsis genome, q-value and percentage of Methylation difference, identification number and TE/Gene description according to Araport11 (06/2016). The values of the % of methylation differences of galls respect to the uninfected roots from the analysis performed in this study (meth.diff) and the independent validation performed (meth.diff, validation) are indicated, as well as their corresponding corrected *P*-values (qvalue; qvalue, validation). MethylC-seq IGV visualization of the methylation patterns from three independent biological replicates of uninfected control roots and galls used in this analysis (RC1-RC3, Gall1-Gall3; black; Material and Methods), and three independent replicates from uninfected control roots and galls used for validation (RC4-6, Gall4-

Gall6; grey; Methods S1), at CHG context, of three representative DMRs (d), AT2TE18860, AT4TE19135 and AT4TE64180 are shown.

Figure S2 – Differentially Expressed Genes (DEGs) in Arabidopsis galls/GCs at 3 d post-infection (dpi) formed by *Meloidogyne javanica* by RNA-seq and microarrays. Number of galls DEGs at 3 dpi (RNA-seq) overlapping the DEGs (microarrays) from 3 dpi galls described in Barcala *et al*. (2010; a) and those overlapping the transcriptome of 3 dpi micro-dissected giant cells DEGs in Barcala *et al*. (2010; b). Classification of DEGs in RNA-seq (**Table S3**) in different categories of MAPMAN (version 3.6.0RC1; Usadel *et al.*, 2009; c). Overrepresented categories are indicated by an asterisk (*) and were obtained using a Wilcoxon Rank Sum Test (Wilcoxon, 1945) and a Benjamini Hochberg (Benjamini & Hochberg, 1995; $P < 0.05$). The category 'metabolism' included: secondary metabolism, amino acid metabolism, major CHO metabolism, minor CHO metabolism, glycolysis, fermentation, Opp, TCA/org. transformation, lipid metabolism, N-metabolism, polyamide metabolism, nucleotide metabolism and C1-metabolism as described in Barcala *et al*. (2010). The Venn diagrams were obtained using VENNY 2.1 (https://bioinfogp.cnb.csic.es/tools/venny/). Red represents induced and green represents repressed genes respect to the control uninfected roots.

Figure S3 – Expression levels of Differentially Expressed Genes in galls at 3 d post-infection (RNA-seq) involved in several epigenetic processes. The genes described for the non-canonical RdDM pathway by Cuerda-Gil & Slotkin (2016) and the list of components listed in Matzke & Mosher (2014) were considered for the comparison. DRM1 was added as it was not originally described in those studies. X-axis, Log_2 of the fold change; and y-axis, abbreviations of genes described in the references previously mentioned. Red represents induced and green represents repressed genes respect to the control uninfected roots.

Figure S4 – Gene Ontology of Differentially Expressed Genes (RNAseq; DEGs) in galls at 3 d post-infection overlapping Differentially Methylated Regions (DMRs). Number of DMRs (genes and promoters) that overlap with DEGs (up- and downregulated, red and green, respectively) classified in different categories following MAPMAN. The DEGs were considered with an adjusted *P*-value cutoff of 0.05. The

represented categories were obtained using a Wilcoxon Rank Sum Test not corrected and no overrepresented categories for a $P < 0.05$ were obtained. Red represents induced and green represents repressed genes respect to the control uninfected roots.

Methods S1. Detailed description of extended methods.

Supplementary videos (see separate files)

Video 1: Col-0 Gall 14 dpi

Video 2: *ddc* Gall 14 dpi

Video 3: *cmt2* Gall 14 dpi

Video 4: *cmt3* Gall 14 dpi

Video 5: *cmt2-cmt3* Gall 14 dpi

Video 6: *ros1* Gall 14 dpi

Video 7*: met1* Gall 14 dpi

Video 8: *drm1/2* Gall 14 dpi

Supplementary Tables (see separate files)

Table S1 – List of primers used on this study for mutant genotyping and Arabidopsis mutants used for functional studies with their corresponding SALK numbers and reference information.

Table S2 – Summary of general data for MethylC-sequencing and processing of galls (three replicates: G1, G2 and G3) induced by *Meloidogyne javanica* and controls (three replicates: RC1, RC2 and RC3) at 3 d post-infection (dpi; a) and at 14 dpi (b). The bisulfite conversion efficiency was calculated using both the chloroplast and the non-methylated λ phage DNA, added to the libraries before sequencing. Number of methylated and unmethylated cytosines by chromosome (Chr), chloroplast and mitochondria and by methylation context (CG, CHG and CHH) at 3 dpi (c) and at 14 dpi (d). Material and Methods on library preparation are detailed in the sheet "Methods".

Table S3 – Summary of general data for RNA-sequencing processing of galls (three replicates: G1, G2 and G3) induced by *Meloidogyne javanica* and controls (three replicates: RC1, RC2 and RC3) at 3 d post-infection. Material and Methods on library preparation are detailed in the sheet "info-methods". Differentially Expressed Genes

(DEGs), (a) List of DEGs, |$\log_2$-fold change| > 0.5 and adjusted *P*-value < 0.01 (b) List of DEGs with adjusted *P*-value < 0.05.

Table S4 – Percentage (a) and number (b) of unique small interfering RNAs (siRNAs) matching genes, promoters and TEs identified in Differentially Methylated Regions (DMRs; methylation difference > 15%) at 3 d post-infection galls. Summary tables with percentages of DMRs based on different classifications (overlapping TEs, overlapping promoters, overlapping promoters/TEs; overlapping promoters/no TEs) are also shown. siRNAs exclusive of Galls (eGall; 21, 22 and 24-nt long), RC (eRC) and present in both (eboth). A similar analysis was performed for Final Genes and Final Promoters defined as those overlapping a Differentially Methylated Region and differentially expressed (adjusted *P*-value < 0.05; **Table S3b**). NA – not available.

Table S5 – Table of general Differentially Methylated Regions (DMRs) at 3 d post-infection (dpi; a) and 14 dpi (b). All the DMRs with q-value < 0.05 identified in this study are classified by chromosome, methylation context, methylation difference of galls *versus* control, start and end position, *P*- and q-values and the genomic regions (genes, promoters and transposable elements or TEs). Galls (three replicates: G1, G2 and G3) induced by *Meloidogyne javanica* and controls (three replicates: RC1, RC2 and RC3). numCs – number of cytosines in the corresponding replicate; numTs – number of thymines in the corresponding replicate. NA – not available.

Table S6 – Percentage of Transposable Elements (TEs) described in the *Arabidopsis thaliana* genome (TAIR10) and Differentially Methylated Regions (DMRs; methylation difference > 15%) matching TEs in Arabidopsis galls induced by *Meloidogyne javanica* (3 and 14 d post-infection) classified in Class I (retrotransposons that function via intermediate RNAs and reverse transcriptase), Class II (DNA-TEs that function via a DNA intermediate and transposase) and different TEs superfamilies. Classification as “Only TEs” and “Promoters/ TEs” are included.

Table S7 – Total number and percentages of Differentially Expressed Genes in galls formed by *Meloidogyne javanica* in *Arabidopsis thaliana* at 3 d post-infection, common with genes showing significant fluctuations with a peak in expression during different phases of the cell division cycle (S, G2, M, G1) accordingly to Menges *et al.*

(2002).

Table S8 – Differentially Expressed Genes (DEGs) classified by categories represented in MAPMAN. Number of up- and downregulated genes at 3 d post-infection in each category. The categories in red were included in ‘Metabolism’. 7395 DEGs out of 7458 were mapped in the 36 main categories, including the highest number in non-assigned.

Table S9 – Differentially Expressed Genes represented in the MAPMAN category ‘microRNAs, natural antisense etc’, in galls at 3 d post-infection.

Table S10 – Genes and promoters that overlapped a Differentially Methylated Region (DMR; methylation difference >15%) and were differentially expressed in galls at 3 days post infection (adjusted *P*-value < 0.05). In red are marked the loci that showed significant methylation differences in both genes and promoters. Galls (three replicates: G1, G2 and G3) induced by *Meloidogyne javanica* and controls (three replicates: RC1, RC2 and RC3). numCs – number of cytosines in the corresponding replicate; numTs – number of thymines in the corresponding replicate. NA – not available.

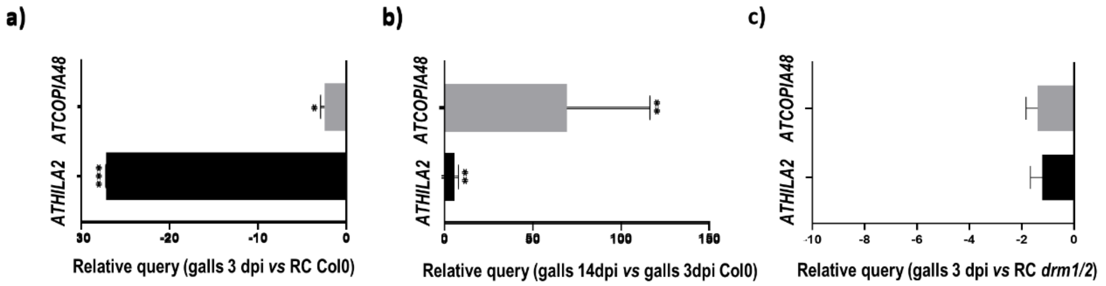

d)

ID	Chr	start	end	qvalue	meth.diff	qvalue (validation)	meth.diff (validation)	TE/Gene
AT2TE18860	2	4504201	4504400	8.51E-06	17.4179298	0.00042907	9.52163648	LTR/Gypsy
AT2TE20815	2	5061201	5061400	0.01040008	17.2264808	0.01701927	8.37955574	LINE/L1
AT2TE10955	2	2381001	2381200	0.03904381	10.7208916	0.04359096	9.25870077	LTR/Gypsy
AT3TE40420	3	9695801	9696000	0.0260167	10.2802255	4.31E-07	10.3371955	LTR/Copia
AT4TE64180	4	13627201	13627400	4.18E-06	8.7241043	0.00042907	10.4831595	LTR/Copia
AT4TE19135	4	4561801	4562000	0.00352485	17.5406108	0.02465635	11.8986979	DNA/En-Spm
AT2G13150	2	5437201	5437400	0.03521666	21.859114	0.02336977	9.46234986	bZIP TF

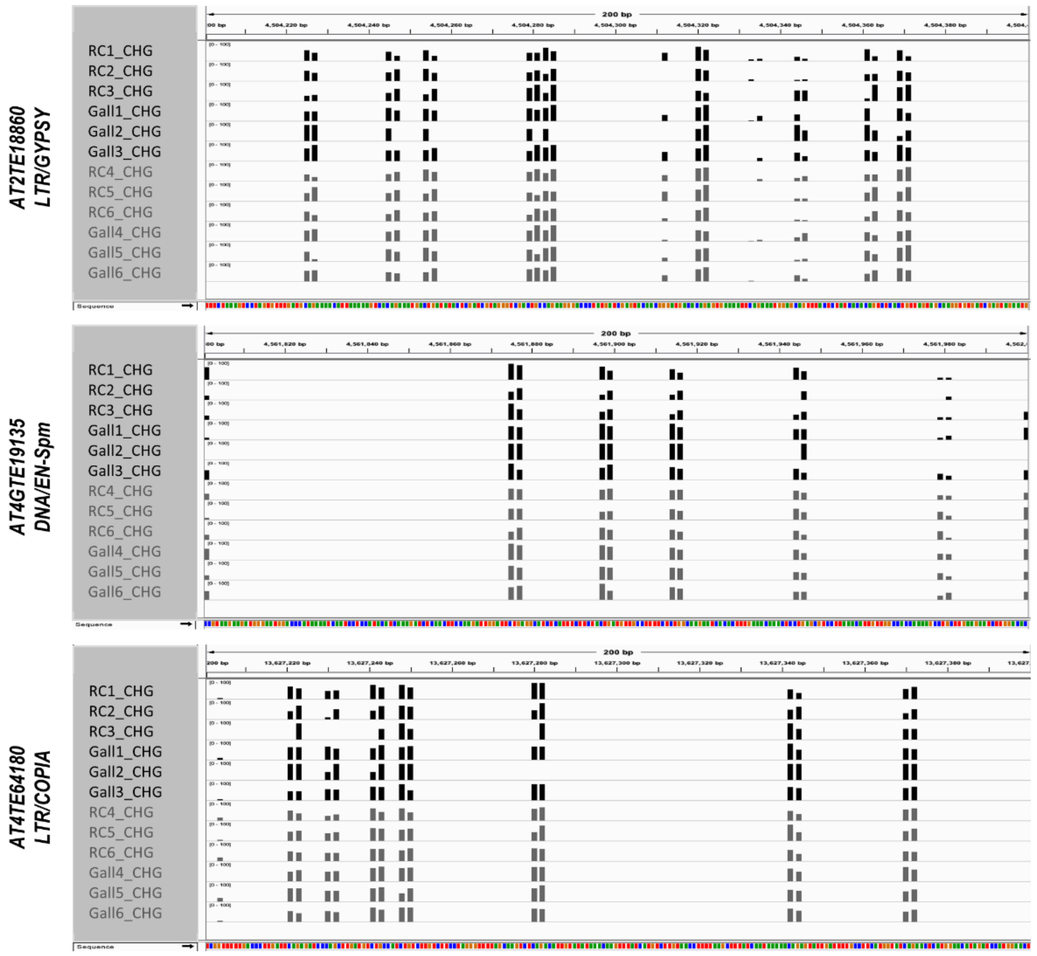

Figure S1 - Expression of Class I Transposon elements (retrotransposons) and validation of MethylC-seq on selected DMRs at 3 d post-infection (dpi; Methods S1). (a) Relative expression levels by quantitative polymerase chain reaction (qPCR) of

ATHILA2 and ATCOPIA48, representatives of retrotransposon superfamilies in Col-0 galls *versus* Col-0 uninfected control roots, at 3 d post-infection (3 dpi), (b) in Col-0 galls at 14 dpi *versus* galls at 3 dpi and (c) in 3 dpi galls *versus* uninfected control roots from the *drm1/2* Arabidopsis background. Three independent biological replicates, with three technical replicates each were performed and normalized to Glyceraldehyde-3-Phosphate Dehydrogenase (GADPH; Ruiz-Ferrer *et al*., 2018). Differences from control values were significant at **P* < 0.05, ***P* < 0.01, and ****P* < 0.001 (two-tailed *t*-test). Values are means ± SE. (d) Validation of MethylC-seq on selected DMRs, at 3 dpi. All DMRs described in this table are classified by chromosome, start and end position on Arabidopsis genome, q-value and percentage of Methylation difference, identification number and TE/Gene description according to Araport11 (06/2016). The values of the % of methylation differences of galls respect to the uninfected roots from the analysis performed in this study (meth.diff) and the independent validation performed (meth.diff, validation) are indicated, as well as their corresponding corrected *P*-values (qvalue; qvalue, validation). MethylC-seq IGV visualization of the methylation patterns from three independent biological replicates of uninfected control roots and galls used in this analysis (RC1-RC3, Gall1-Gall3; black; Material and methods), and three independent replicates from uninfected control roots and galls used for validation (RC4-6, Gall4-Gall6; grey; Methods S1), at CHG context, of three representative DMRs (d), AT2TE18860, AT4TE19135 and AT4TE64180 are shown.

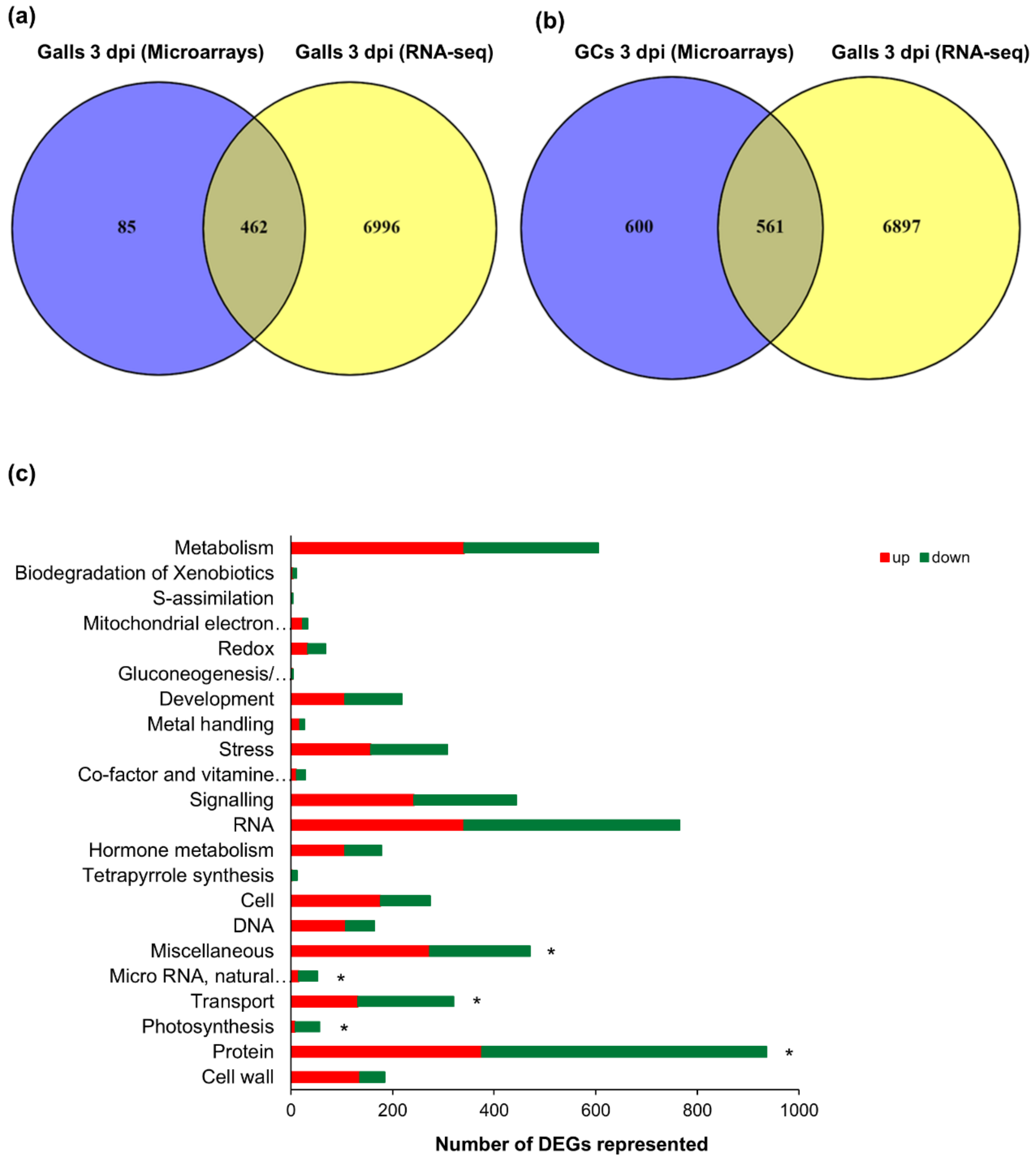

Figure S2 – Differentially Expressed Genes (DEGs) in Arabidopsis galls/GCs at 3 d post-infection (dpi) formed by *Meloidogyne javanica* by RNA-seq and microarrays. Number of galls DEGs at 3 dpi (RNA-seq) overlapping the DEGs (microarrays) from 3 dpi galls described in Barcala *et al.* (2010; a) and those overlapping the transcriptome of 3 dpi micro-dissected giant cells DEGs in Barcala *et al.* (2010; b). Classification of DEGs in RNA-seq (**Table S3**) in different categories of MAPMAN (version 3.6.0RC1; Usadel *et al.*, 2009; c). Overrepresented categories are indicated by an asterisk (*) and were obtained using a Wilcoxon Rank Sum Test (Wilcoxon, 1945) and a Benjamini Hochberg (Benjamini & Hochberg, 1995; $P < 0.05$). The category

‘metabolism’ included: secondary metabolism, amino acid metabolism, major CHO metabolism, minor CHO metabolism, glycolysis, fermentation, Opp, TCA/org. transformation, lipid metabolism, N-metabolism, polyamide metabolism, nucleotide metabolism and C1-metabolism as described in Barcala *et al*. (2010). The Venn diagrams were obtained using VENNY 2.1 (https://bioinfogp.cnb.csic.es/tools/venny/). Red represents induced and green represents repressed genes respect to the control uninfected roots.

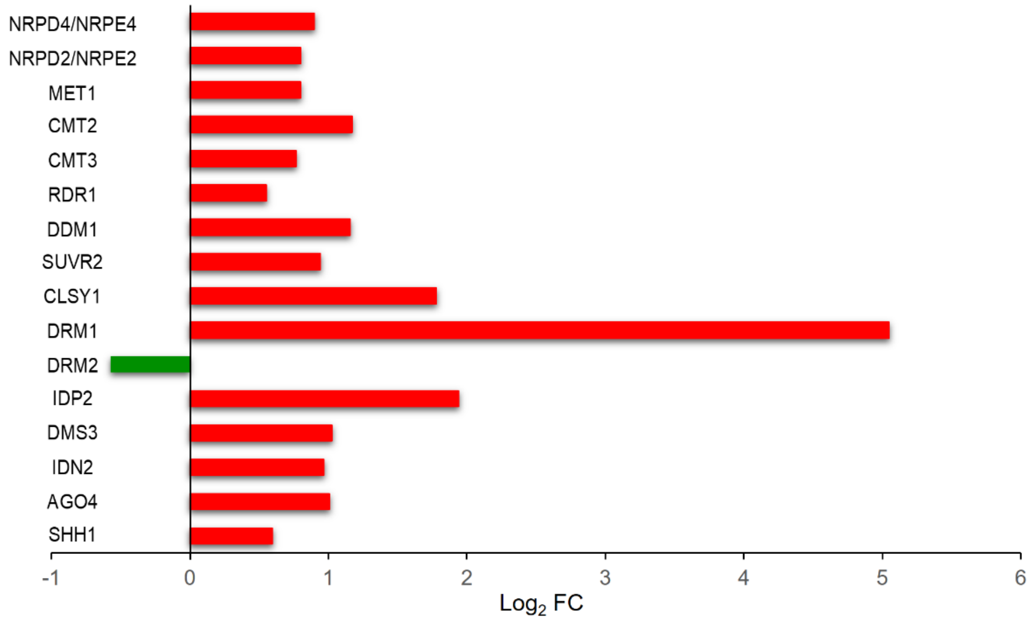

Figure S3 – Expression levels of Differentially Expressed Genes in galls at 3 d post-infection (RNA-seq) involved in several epigenetic processes. The genes described for the non-canonical RdDM pathway by Cuerda-Gil & Slotkin (2016) and the list of components listed in Matzke & Mosher (2014) were considered for the comparison. DRM1 was added as it was not originally described in those studies. X-axis, Log_2 of the fold change; and y-axis, abbreviations of genes described in the references previously mentioned. Red represents induced and green represents repressed genes respect to the control uninfected roots.

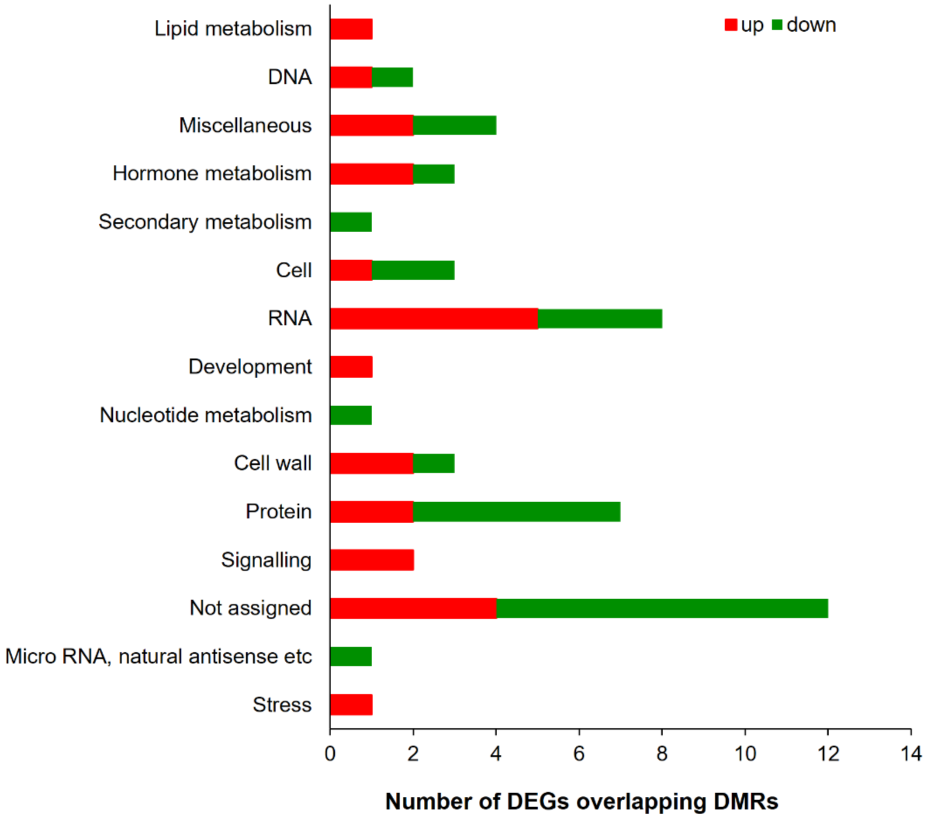

Figure S4 – Gene Ontology of Differentially Expressed Genes (RNAseq; DEGs) in galls at 3 d post-infection overlapping Differentially Methylated Regions (DMRs). Number of DMRs (genes and promoters) that overlap with DEGs (up- and downregulated, red and green, respectively) classified in different categories following MAPMAN. The DEGs were considered with an adjusted *P*-value cutoff of 0.05. The represented categories were obtained using a Wilcoxon Rank Sum Test not corrected and no overrepresented categories for a $P < 0.05$ were obtained. Red represents induced and green represents repressed genes respect to the control uninfected roots.

METHODS S1

RNA and DNA extraction and purification

Only galls formed in the main primary roots were hand dissected together with equivalent root segments from uninfected plants to avoid epigenetic biases due to differences between the primary root, directly derived from embryogenesis, and the lateral roots, derived from post-embryogenic new organogenesis. For Arabidopsis, a total of 10 independent experiments were pooled approximately in triplets for each biological replicate, forming three independent biological samples per treatment and used for simultaneous total RNA and genomic DNA extraction. Each biological replicate contained at least 250 hand dissected galls (from 200-300 independent plants) and 400-900 root segments from uninfected roots (from 400-900 plants) at 14 and 3 dpi, respectively, following Silva *et al*. (2019).

High-throughput MethylC-sequencing (MethylC-seq) library preparation and data processing

Validation focused on some representative DMRs was performed by WGBS with 6 new independent DNA libraries, processed in the same way as described in Material and Methods. Three independent biological replicates of uninfected root segments and three of hand dissected galls at 3 dpi were collected as described in the general Material and Methods (Fig. S4d). Basically, the libraries were prepared using the EZ DNA Methylation-Gold Kit (Zymo Research, Freiburg, Germany) following the library protocol Accel-NGS Methyl-Seq DNA Library Kit for Illumina Platforms by Macrogen Inc. (South Korea) and sequenced using an Illumina® HiSeq X PE150 (San Diego, California, USA).

High-throughput RNA-sequencing (RNA-seq) library preparation and data processing

RNA-sequencing raw data and detailed data processing is available in GEO accession number GSE155171. The reads were firstly checked for quality using FastQC (version 0.11.8). Trimming was then performed using BBDuk (version 38.39). Trimmed reads were mapped to the combined *Arabidopsis thaliana* (TAIR10) and *M. javanica* (Assembly GCA_900003945.1; Blanc-Mathieu *et al*., 2017) genomes using STAR (version 2.7.0d; Dobin *et al*., 2013). HTSeq (version 0.11.2) was used to produce the

count tables used for the differentially expressed analysis.

The pipeline used for the sRNA analysis was the same as described in Ruiz-Ferrer *et al*. (2018) for the group of rasiRNAs; but in this study the analysis was performed on the full set of sRNAs from the 6 sRNAs libraries (Table S4).

Total RNA isolation for q-PCR analysis

Three new independent biological replicates of Col-0 control root segments from uninfected plants and galls from plants infected with *M. javanica* were hand dissected at 3 dpi, according to Barcala *et al*. (2010). Total RNA extraction was performed according to Silva *et al*. (2019). One microgram of total RNA from each sample was used for cDNA synthesis with a High-Capacity cDNA Reverse Transcription Kit (Thermo Fisher Scientific, Waltham, Massachusetts, USA) with selected primers (Table S1). The cDNA was used at 50 ng per reaction for ATHILA2 and ATCOPIA48 TEs (class I). Relative gene expression was determined using the ΔΔCt method and a LightCycler® 480 II machine (Roche, Indianapolis, IN, USA). Undetectable transcripts were set to a value of 40 PCR cycles in order to perform the statistics (McCall *et al*., 2014). Values from three independent biological replicates, with three technical replicates each were used; all were normalized to the Glyceraldehyde-3-Phosphate Dehydrogenase (GADPH) internal control (Barcala *et al*., 2010; Ruiz-Ferrer *et al*., 2018). Differences from control values were significant at *$P < 0.05$, **$P < 0.01$, ***$P < 0.001$ (Student's *t*-test comparing ΔCt values, $P < 0.05$).

References

Barcala M, García A, Cabrera J, Casson S, Lindsey K, Favery B, García-Casado G, Solano R, Fenoll C, Escobar C. **2010**. Early transcriptomic events in microdissected Arabidopsis nematode-induced giant cells. *Plant Journal* **61**: 698–712.

Benjamini Y, Hochberg Y. **1995**. Controlling the false discovery rate: A practical and powerful approach to multiple testing. *Journal of the Royal Statistical Society: Series B (Methodological)* **57**: 289–300.

Blanc-Mathieu R, Perfus-Barbeoch L, Aury JM, Da Rocha M, Gouzy J, Sallet E, Martin-Jimenez C, Bailly-Bechet M, Castagnone-Sereno P, Flot J, *et al*. 2017. Hybridization and polyploidy enable genomic plasticity without sex in the most devastating plant-parasitic nematodes. *PLoS Genetics* **13**: e1006777.

Cuerda-Gil D, Slotkin RK. **2016**. Non-canonical RNA-directed DNA methylation. *Nature Plants* **2**: 1–8.

Dobin A, Davis CA, Schlesinger F, Drenkow J, Zaleski C, Jha S, Batut P, Chaisson M, Gingeras TR. 2013. STAR: Ultrafast universal RNA-seq aligner. *Bioinformatics* **29**: 15–21.

Matzke MA, Mosher RA. **2014**. RNA-directed DNA methylation: An epigenetic pathway of increasing complexity. *Nature Reviews Genetics* **15**: 394–408.

McCall MN, McMurray HR, Land H and Almudevar A. 2014. On non-detects in qPCR data. *Bioinformatics* **30:** 2310–2316.

Menges M, Hennig L, Gruissem W, Murray JAH. **2002**. Cell cycle-regulated gene expression in Arabidopsis. *Journal of Biological Chemistry* **277**: 41987–42002.

Ruiz-Ferrer V, Cabrera J, Martinez-Argudo I, Artaza H, Fenoll C, Escobar C. **2018**. Silenced retrotransposons are major rasiRNAs targets in Arabidopsis galls induced by *Meloidogyne javanica*. *Molecular Plant Pathology* **19**: 2431–2445.

Silva AC, Ruiz-Ferrer V, Martínez-Gómez Á, Barcala M, Fenoll C, Escobar C. **2019**. All in one high quality genomic DNA and total RNA extraction from nematode induced galls for high throughput sequencing purposes. *Frontiers in Plant Science* **10**: 657.

Usadel B, Poree F, Nagel A, Lohse M, Czedik-Eysenberg A, Stitt M. **2009**. A guide to using MapMan to visualize and compare Omics data in plants: A case study in the crop species, Maize. *Plant, Cell and Environment* **32**: 1211–1229.

Wilcoxon F. **1945**. Individual comparisons by ranking methods. In: Kotz S, Johnson NL, eds. *Breakthroughs in Statistics. Springer Series in Statistics.* New York,

NY, USA: Springer, 196–202.
